# The Effects of Sika Deer Antler Peptides on 3T3-L1 Preadipocytes and C57BL/6 Mice via Activating AMPK Signaling and Gut Microbiota

**DOI:** 10.3390/molecules30051173

**Published:** 2025-03-06

**Authors:** Tong Sun, Zezhuang Hao, Fanying Meng, Xue Li, Yihua Wang, Haowen Zhu, Yong Li, Yuling Ding

**Affiliations:** 1 School of Pharmaceutical Sciences, Changchun University of Chinese Medicine, Changchun 130117, China; 22203089218@stu.ccucm.edu.cn (T.S.); 23203070601@stu.ccucm.edu.cn (Z.H.); 22203089214@stu.ccucm.edu.cn (F.M.); 22203089213@stu.ccucm.edu.cn (X.L.); 24204892085@stu.ccucm.edu.cn (Y.W.); 2 College of Life Sciences, University of Camerino, 62032 Camerino, Macerata Province, Italy; haowen.zhu@studenti.unicam.it

**Keywords:** sika deer, velvet antler peptides, fat accumulation, gut microbiota, AMPK signaling pathway

## Abstract

(1) Background: To explore the anti-obesity effects and mechanisms of sika deer velvet antler peptides (sVAP) on 3T3-L1 preadipocytes and in high-fat diet (HFD)-induced obese mice. (2) Methods: sVAP fractions of different molecular weights were obtained via enzymatic hydrolysis and ultrafiltration. Their anti-lipid effects on 3T3-L1 cells were assessed with Oil Red O staining. The optimal fraction was tested in HFD-induced obese C57BL/6 mice to explore anti-obesity mechanisms. Peptide purification used LC-MS/MS, followed by sequence analysis and molecular docking for activity prediction. (3) Results: The peptide with the best anti-obesity activity was identified as sVAP-3K (≤3 kDa). sVAP-3K reduced lipid content and proliferation in 3T3-L1 cells, improved lipid profiles and ameliorated adipocyte degeneration in HFD mice, promoted the growth of beneficial gut microbiota, and maintained lipid metabolism. Additionally, sVAP-3K activated the AMP-activated protein kinase (AMPK) signaling pathway, regulating adipogenic transcription factors. sVAP-3K exhibited ten major components (peak area ≥ 1.03 × 10^8^), with four of the most active components being newly discovered natural oligopeptides: RVDPVNFKL (*m/z* 363.21371), GGEFTPVLQ (*m/z* 474.24643), VDPENFRL (*m/z* 495.25735), and VDPVNFK (*m/z* 818.44043). (4) Conclusion: This study identifies four novel oligopeptides in sVAP-3K as key components for anti-obesity effects, offering new evidence for developing natural weight-loss drugs from sika deer velvet.

## 1. Introduction

According to estimates from the World Health Organization on the global population with a BMI ≥ 25 kg m^−2^, indicating overweight and obesity, the proportion of the world’s population that is overweight or obese is projected to increase from 38% in 2020 to over 50% by 2035 [1]. Numerous studies have shown that obesity increases the incidence of type II diabetes, cardiovascular diseases, fatty liver, and cancer [2,3,4]. The fundamental cause of obesity is an imbalance between energy intake and energy expenditure. Current strategies for the prevention and treatment of obesity are categorized into four types, which include reducing food intake, blocking nutrient absorption, increasing thermogenesis, and regulating energy metabolism or storage [5]. Traditionally, anti-obesity strategies have focused on dietary and lifestyle changes, such as caloric restriction and enhanced exercise [6,7]. However, it is challenging for obese individuals to maintain these strategies, prompting the search for new treatment methods to combat obesity. In this context, recent years have seen a broad interest in the potential of amino acid derivatives for the prevention and/or treatment of obesity [8,9,10].

As a key regulator of adipogenesis, the AMP-activated protein kinase (AMPK) signaling pathway not only maintains cellular energy balance and physiological metabolism [11], but can also activate the downregulation of transcription factors such as peroxisome proliferator-activated receptor CCAAT/enhancer-binding protein-α (C/EBPα) [12]. Upon activation, C/EBPα upregulate many genes in the adipocyte phenotype, including glycerol phosphate dehydrogenase, insulin receptor, and fatty acid-binding protein [1]. Therefore, inhibiting adipogenesis or adipocyte differentiation could be an effective strategy for the treatment or prevention of obesity and related diseases.

There is also a close relationship between the gut microbiota and HFD [13]. The gut microbiota can affect the host’s energy and lipid metabolism, increasing the risk of metabolic diseases and intestinal permeability, and inducing inflammation. Dietary intake is a significant factor influencing the biochemical composition of the gut microbiota, which can lead to changes in bacterial cell types, composition, and activity. Notably, a decrease in beneficial bacteria and an increase in opportunistic pathogens can lead to lipid metabolism disorders [14,15]. For instance, long-term consumption of HFD can lead to dysbiosis of the gut microbiota [16]. Therefore, treatments that block lipid accumulation (Lip-acc) and promote energy metabolism by regulating these metabolic factors can impact lipid levels.

Velvet antler is a typical animal-derived traditional medicine that has been used for over 2000 years. In many countries in Asia, particularly in China, the antlers from sika deer and red deer are legally recognized sources of velvet antler [17]. Previous studies have reported that the enzymatic hydrolysis products of red deer velvet antler exhibit anti-obesity effects [18]; however, there are no reports on the anti-obesity activity of sika deer velvet antler. Traditional methods for extracting bioactive components from velvet antler typically involve slow boiling in hot water. However, this method is contentious due to the extremely low recovery rate of water-soluble components during the extraction process. Over the past decade, enzyme-assisted extraction technology has been successfully applied to extract numerous bioactive natural products from various organisms, including proteins. Partially digestive proteases and commercial proteases (pepsin, trypsin, chymotrypsin, dispase, alcalase, protamex) are commonly used because of their high specificity, mild reaction conditions, environmental friendliness, and ease of inactivation or control [19,20,21,22,23].

Therefore, the aim of this study is to investigate the effects of velvet antler hydrolysates on Lip-acc and the inhibition of adipocyte differentiation in 3T3-L1 cells. Additionally, we examine the impact of these hydrolysates on obesity by assessing relevant parameters, histology, and changes in gut microbiota composition in high-fat diet-induced obese mice.

## 2. Results

### 2.1. Screening the Optimal Hydrolysate Using Differentiated 3T3-L1 Preadipocytes

**Figure 1:**
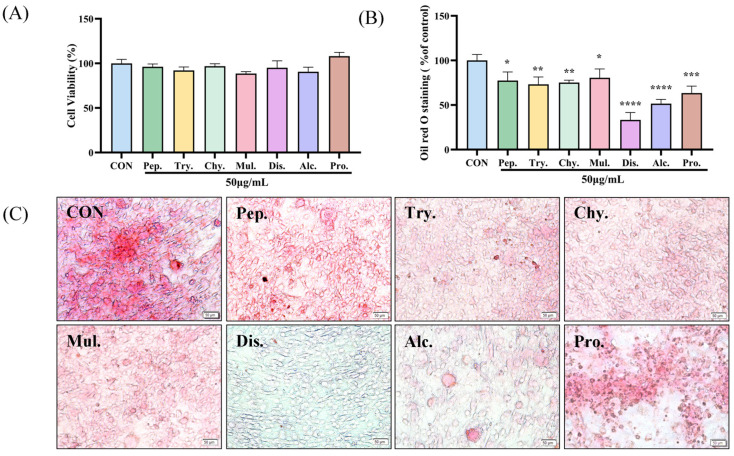
Screening the optimal deer antler hydrolysate using 3T3-L1 preadipocytes. (**A**) Cytotoxicity assay of different hydrolysate at 50 μg/mL. (**B**) Quantification of the lipid accumulation in 50 μg/mL sVAP-treated and non-treated (control) adipocytes after Oil Red O elution. (**C**) Intracellular lipid accumulation in 3T3-L1 adipocytes after completion of the differentiation process (50 μm = 20×). Microscopic images of adipocytes stained with Oil Red O. Compared to the CON group: * *p* < 0.05, ** *p* < 0.01, *** *p* < 0.001, and **** *p* < 0.0001. Pep.—pepsin; Try.—trypsin; Chy.—chymotrypsin; Mul.—multi-enzyme; Dis.—dispase; Alc.—alcalase; Pro.—protamex.

The administered concentration was determined using the MTT assay, The MTT method was used to determine the sample concentration, and at a concentration of 50 μg/mL, seven hydrolysates showed no toxicity to cells, as shown in Figure 1. Seven hydrolysates were applied to differentiation-induced 3T3-L1 preadipocytes at a concentration of 50 μg/mL. After staining with ORO, the dispase hydrolysates (DH) showed the strongest inhibitory activity, while the alcalase and protamex hydrolysates were second only to the DH in terms of their ability to inhibit adipose growth.

### 2.2. Different Molecular Weights of sVAP Inhibit the Differentiation of 3T3-L1 Preadipocytes into Adipocytes

**Figure 2:**
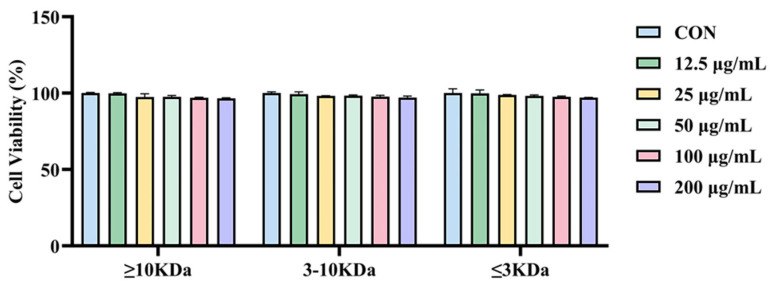
Cytotoxicity assay of sVAP with different molecular weights on 3T3-L1 cells.

**Figure 3:**
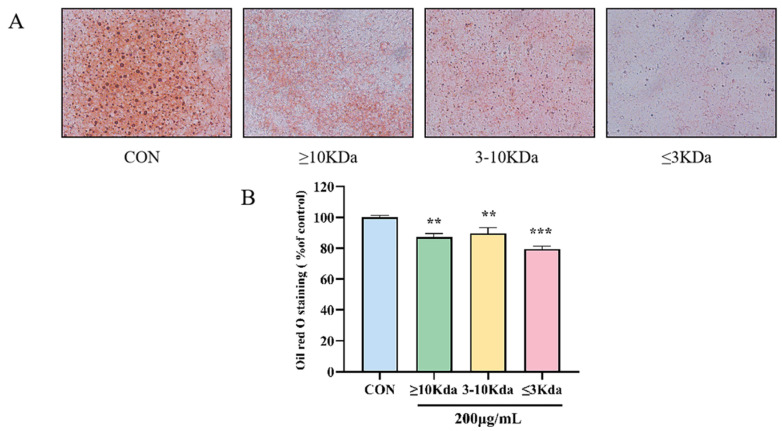
(**A**) Fix cells and stain with ORO (50 μm). (**B**) Dissolve the stained lipid droplets in isopropanol and quantify intracellular Lip–acc. Compared to the CON group: ** *p* < 0.01, and *** *p* < 0.001.

In differentiated 3T3-L1 cells, it was observed through ORO staining that the accumulation of lipid droplets in sVAP-treated cells was significantly lower than the CON group. Firstly, the MTT assay was used to determine the effect of different molecular weights of sVAP at different concentrations on cell viability 48 h after induction. Due to the fact that sVAP treatment with different molecular weights up to 200 μg/mL did not affect cell viability, as shown in Figure 2, the ORO staining experiment was conducted at a concentration of 200 μg/mL. To investigate the inhibitory effect of sVAP with different molecular weights on intracellular Lip-acc, ORO staining was performed. The differentiation of 3T3-L1 cells leads to Lip-acc, while treatment with sVAP of different molecular weights reduces this accumulation. Among them, sVAP-3K can most effectively inhibit Lip-acc, as shown in Figure 3A. So, we named the portion of DH with a molecular weight of ≤3 KDa—sVAP–3K.

### 2.3. sVAP-3K Reduced Weight Gain and Adipose Tissue Weight in HFD Mice

**Figure 4:**
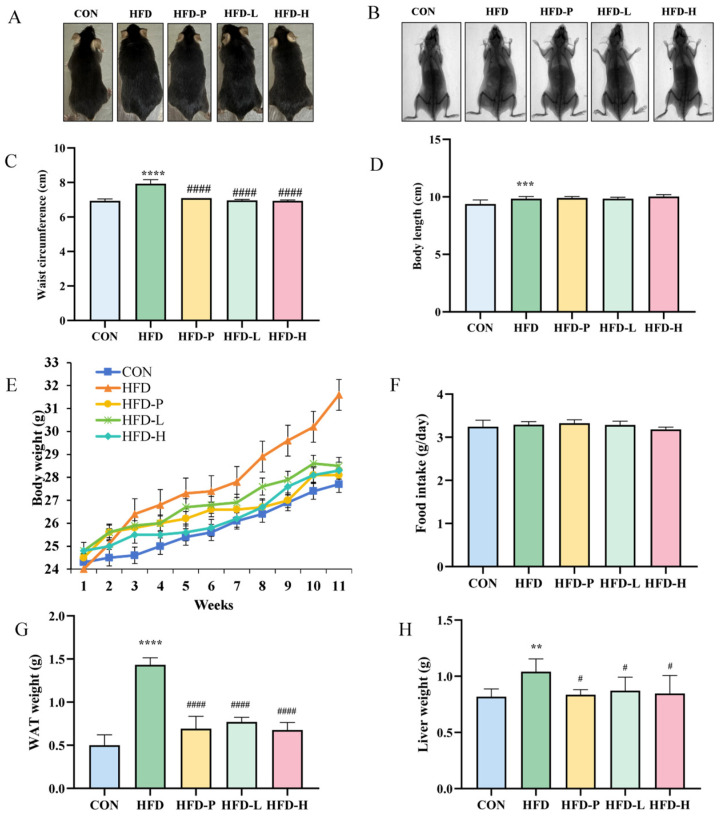
The effect of oral administration of sVAP-3K on HFD-induced weight gain and dietary intake in C57BL/6 mice. Administer sVAP (150 or 300 mg/kg) to HFD-induced C57BL/6 mice five times a week for 11 weeks. (**A**) Display of representative mice from each group at the end of week 11. (**B**) Image confirming the body adipose using CT method. (**C**) Mouse abdominal circumference data. (**D**) Mouse body length data. (**E**) The weight of mice was measured every week. (**F**) The average weekly food intake of each group. (**G**,**H**) Measurements of abdominal fat and liver weight. Compared to the CON group: ** *p* < 0.01, *** *p* < 0.001, and **** *p* < 0.0001. Compared to the HFD group: # *p* < 0.05 and #### *p* < 0.0001.

Based on in vitro data, a HFD mouse model was used for in vivo experiments to validate the anti-obesity effect of sVAP-3K. The effect of sVAP-3K on mouse body shape is shown in Figure 4A,B. As shown in Figure 4C,D, during the 11-week experimental period, the HFD group showed a significant increase in body weight compared to the CON group. The weight gain of the HFD-L group tends to plateau from week 5 onwards, while the HFD-H group effectively reduced body weight from the third week. Compared with the HFD group, sVAP-3K administration significantly reduced body weight. However, sVAP-3K intervention had no significant effect on energy intake in HFD mice. In addition, as shown in Figure 4E,F, HFD increased abdominal fat and liver weight, while sVAP-3K administration significantly reduced abdominal fat and liver weight. On the contrary, the body adipose of the sVAP-3K (150 and 300 mg/kg) treatment groups was significantly reduced. The research results show that HFD can induce obesity and liver tissue adipose accumulation in mice, and long-term consumption of sVAP-3K can slow down or improve obesity symptoms in mice.

### 2.4. sVAP-3K Improved Serum Glucose Levels in HFD Mice

**Figure 5:**
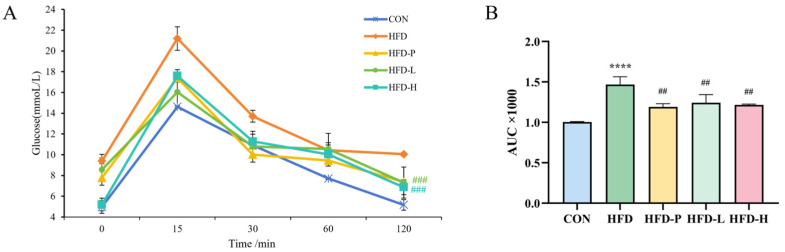
The blood glucose levels and (**A**) offline curve area AUC of (**B**) different groups of C57BL/6 mice at 0, 15, 30, 60, and 120 min after ingestion of 20% glucose. Compared to the CON group: **** *p* < 0.0001. Compared to the HFD group: ## *p* < 0.01 and ### *p* < 0.001.

Due to the induction of dyslipidemia by HFD, we analyzed the blood glucose tolerance levels and serum metabolic indicators in each group. As shown in Figure 5A, high levels of blood glucose intolerance were observed in the HFD group, and oral administration of sVAP-3K significantly increased blood glucose tolerance levels. After oral administration of glucose, the blood glucose levels of all groups of mice rapidly increased, reaching a peak, and then gradually decreased. At the 120 min time points, the blood glucose levels of mice in the HFD-L and HFD-H groups were significantly lower than those in the HFD group, indicating a positive effect of sVAP-3K on glucose metabolism in mice. As shown in Figure 5B, compared with the HFD group, the blood glucose AUC of mice in CON, HFD-P, HFD-L, and HFD-H groups significantly decreased. The test results indicate that HFD can cause impaired glucose tolerance in C57BL/6 mice, and long-term gavage of sVAP-3K can effectively improve glucose tolerance in mice.

### 2.5. sVAP-3K Improved Serum Biochemical Parameters in HFD Mice

**Figure 6:**
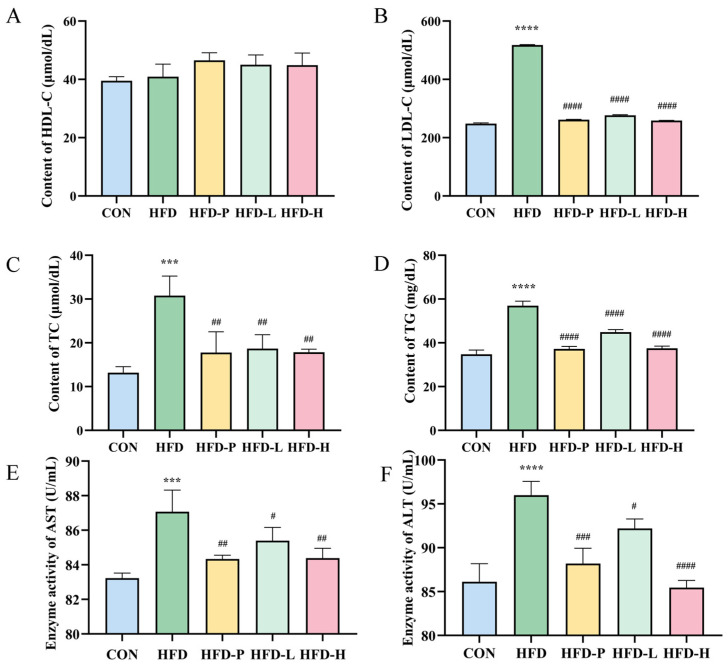
(**A**) High-density lipoprotein cholesterol content. (**B**) Low-density lipoprotein cholesterol. (**C**) Total cholesterol content. (**D**) Triglyceride levels. (**E**) Aspartate transaminase. (**F**) Alanine transaminase. Compared to the CON group: *** *p* < 0.001 and **** *p* < 0.0001. Compared to the HFD group: # *p* < 0.05, ## *p* < 0.01, ### *p* < 0.001, and #### *p* < 0.0001.

The trend of changes in mouse serum biochemical indicators is shown in Figure 6. Compared with the HFD group, sVAP-3K significantly reduced the serum levels of LDL, TC, and TG induced by HFD, as shown in Figure 6B–D. Meanwhile, there was no significant change in HDL levels after sVAP-3K administration, as shown in Figure 6A. In addition, by measuring the serum levels of AST and ALT in C57BL/6 mice fed with HFD, as shown in Figure 6E,F, we found that sVAP-3K has a protective effect on liver function impairment and effectively reduces blood lipid levels in mice by HFD-induced.

### 2.6. sVAP-3K Reduced Adipogenesis in Abdominal Fat Tissue and Liver of HFD Mice via the AMPK Signaling Pathway

**Figure 7:**
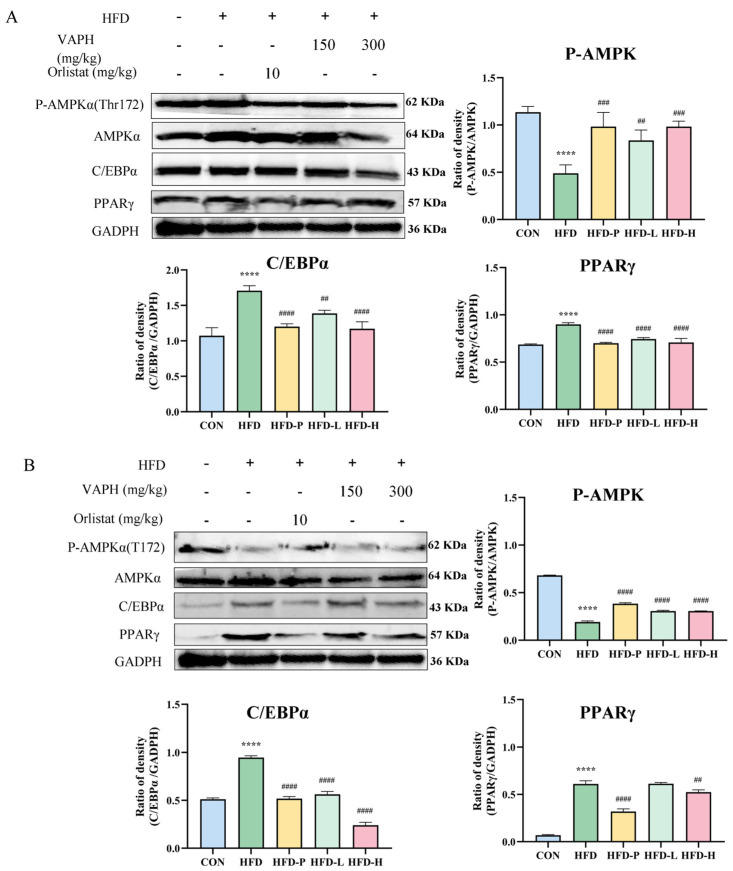
(**A**) The effect of oral sVAP−3K on protein expression in liver tissue of HFD mice. (**B**) The effect of oral sVAP−3K on protein expression in abdominal fat tissue of HFD mice. P−AMPK and lipogenesis-related proteins were evaluated by Western blotting using specific protein antibodies. GADPH protein is used as an internal control. Compared to the CON group: **** *p* < 0.0001. Compared to the HFD group: ## *p* < 0.01, ### *p* < 0.001, and #### *p* < 0.0001.

Through Western blot analysis, it was elucidated that sVAP-3K has an anti-obesity effect in HFD-induced C57BL/6 obese mice. Consistent with in vitro data, administration of sVAP-3K upregulated AMPK activation in liver tissue (Figure 7A) and abdominal fat (Figure 7B) induced by HFD (The original image of the protein band can be found in the Supplementary Materials: Figures S1–S9). sVAP-3K administration significantly reduced the protein expression of the adipogenesis-related transcription factor C/EBPα and PPARγ upregulated by HFD. In mice fed with HFD, sVAP-3K significantly reduced body weight, fat tissue weight, and fat content in tissues. The protein expression of C/EBPα was increased in abdominal fat and liver tissue, while sVAP-3K reduced the expression levels of these transcription factors. Moreover, in the group of mice induced by HFD, the phosphorylation of AMPK was downregulated, and sVAP-3K administration ameliorated AMPK phosphorylation. Therefore, the therapeutic effect of sVAP-3K might be related to the AMPK pathway.

### 2.7. sAP-3K Reduced the Size of Abdominal Adipocytes and Hepatic Lip-acc in HFD Mice

**Figure 8:**
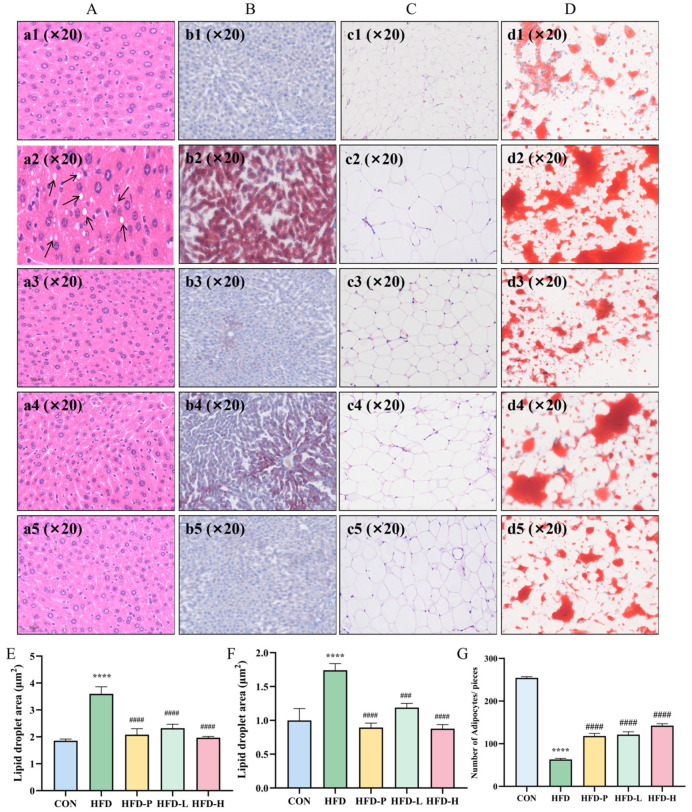
Pathological sections of C57BL/6 mouse liver and adipose tissue. (**A**) HE staining results of mice liver tissue. (**B**) ORO staining results of mice liver tissue. (**C**) HE staining results of abdominal fat tissue in mice. (**D**) The results of abdominal fat ORO staining in mice. (**E**,**F**) The effects of sVAP-3K on adipocytes in the liver and abdominal fat tissue of mice. (**E**) Liver ORO value. (**F**) Fat ORO value. (**G**) The number of adipocytes in each group per equal area. Compared to the CON group: **** *p* < 0.0001. Compared to the HFD group: ### *p* < 0.001, and #### *p* < 0.0001. a/b/c/d1—CON group; a/b/c/d2—HFD group; a/b/c/d3—HFD-P group; a/b/c/d4—HFD-L group; a/b/c/d5—HFD-H group.

The effects of sVAP-3K on liver tissue and abdominal fat tissue of HFD-induced obesity mice were observed using HE staining and ORO staining methods. The histological analysis of liver tissue showed that sVAP-3K administration significantly reduced HFD-induced Lip-acc (Figure 8). In abdominal fat tissue, the diameter of adipocytes in the HFD group is larger than that in the CON group. However, the application of sVAP-3K significantly reduced the enlarged adipocytes (Figure 8). sVAP-3K reduced the size and Lip-acc of adipocytes in abdominal fat and liver tissue, respectively.

### 2.8. Gut Microbiota

#### 2.8.1. Community Diversity Analysis

**Figure 9:**
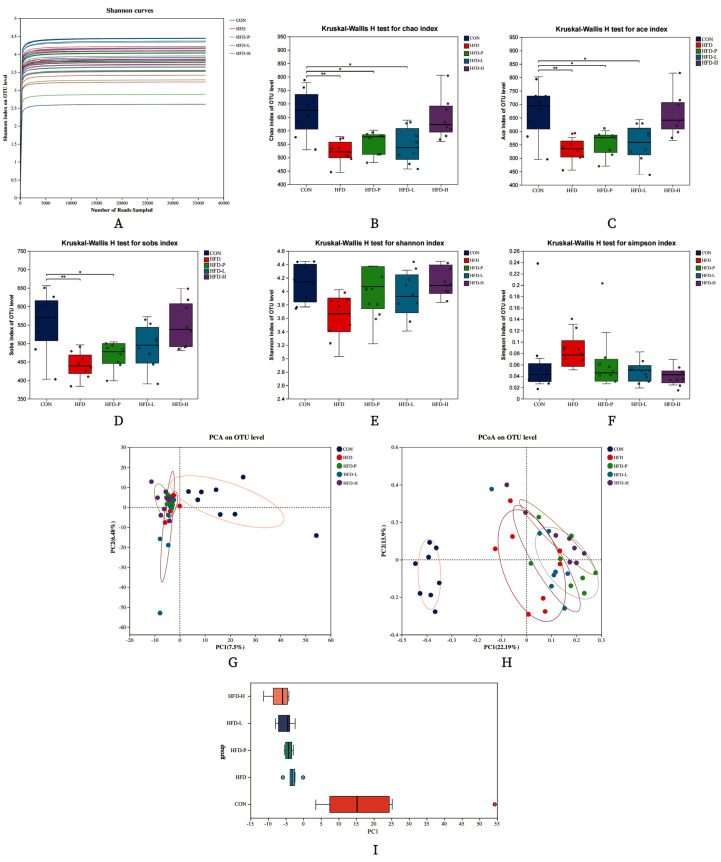
Analysis of gut flora diversity of sVAP−3K on HFD mice. (**A**) Dilution curve. (**B**) Chao index. (**C**) Ace index. (**D**) Sobs index. (**E**) Shannon index. (**F**) Simpson index. (**G**) PCA diagram. (**H**) PCoA diagram. (**I**) PC1 diagram. Compared to the CON group: * *p* < 0.05, ** *p* < 0.01.

The rarefaction curve indicates that the sequencing volume of the samples was adequate, with sufficient data, and the sequencing data were reasonable, as shown in Figure 9A. The diversity and abundance of microbial species are represented by Alpha diversity analysis, depicted in Figure 9B–F. The results show that the community diversity of the HFD group was lower than that of the CON group. The overall effectiveness of the treatment groups was significantly higher than that of the control group.

Beta diversity is mainly used to evaluate the structure and composition of different gut microbiotas, which is reflected by PCA and PCoA. From the PCA plot (where PC1 and PC2 account for 7.5% and 6.48% of the total variance in the gut microbiota structure of mice, respectively), it can be observed that there are differences in the composition structure of the gut microbiota between the CON group and the HFD, HFD-P, HFD-L, and HFD-H groups, as shown in Figure 9G–I. The closer the distance reflected in the PCA plot, the more similar the species composition of the samples. Compared to the CON group, there were significant differences in species composition between the two groups, and their composition was not similar. After treatment, the species composition of the treated groups did not differ significantly from the CON group, and the similarity in composition increased, as shown in Figure 9I.

#### 2.8.2. Community Composition Analysis

**Figure 10:**
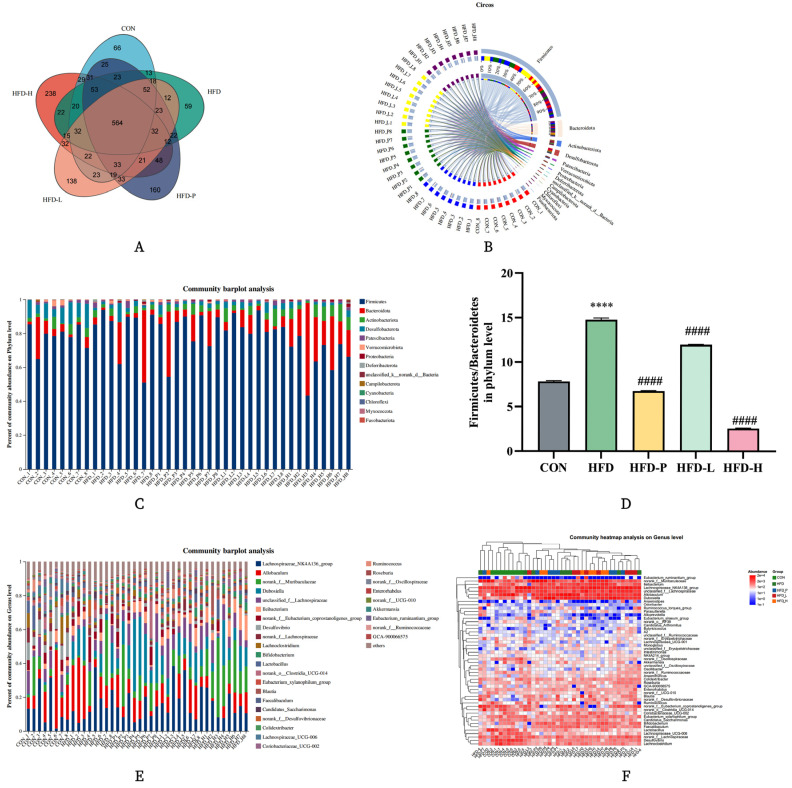
Analysis of community composition in each group. (**A**) Venn diagram. (**B**) Circos diagram. (**C**) Distribution at the level of the phylum. (**D**) The ratio of F/B in each group. (**E**) Distribution at the level of the genus. (**F**) Community heatmap analysis on genus level. Compared to the CON group: **** *p* < 0.0001. Compared to the HFD group: #### *p* < 0.0001.

The Venn diagram shows the shared OTU values among groups, indicating that the HFD-H group is significantly higher than the others, suggesting an increase in species richness within the gut microbiota of the HFD-H group, as shown in Figure 10A. The community Circos diagram reflects the distribution proportion of dominant species in different samples and analyzes the dominant taxa. The phyla Firmicutes and Bacteroidetes are relatively abundant and constitute the dominant taxa in all groups, as depicted in Figure 10B.

The bar chart of the community visually illustrates what types of microorganisms are present in each sample at the phylum level and the relative abundance of each microorganism within the samples. At both the phylum and genus levels, four main phyla were detected: Firmicutes, Bacteroidota, Actinobacteriota, and Desulfobacterota, with Firmicutes and Bacteroidota being the dominant microbial groups, occupying a large proportion. Compared to the CON group, the abundance of Firmicutes in the HFD group was lower, while the abundance of Bacteroidetes was higher, as shown in Figure 10C,D. sVAP-3K significantly inhibited the increase in Firmicutes and the decrease in Clostridia. By observing the Firmicutes/Bacteroidetes ratio (F/B), it was found that the HFD group showed a downward trend, which could be reversed in the treated groups, with the HFD-H group having the lowest ratio, as illustrated in Figure 10E,F.

#### 2.8.3. LEfSe Analysis

**Figure 11:**
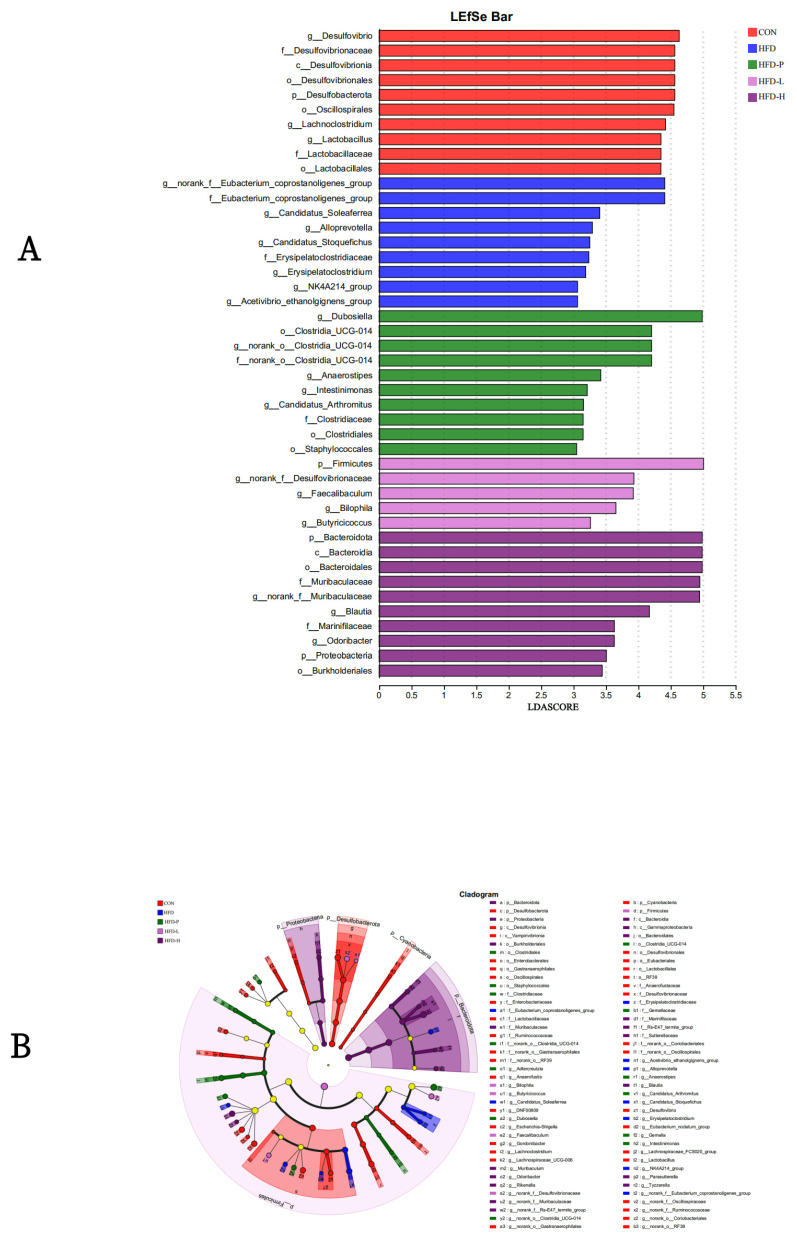
LEfSe analysis: (**A**) LDA score plot and (**B**) LEfSe clade evolutionary diagram.

LEfSe analysis was used to identify the specificity of the gut microbiota in each group (LDA > 4), as shown in Figure 11A,B. In the CON group, six categories were significantly affected (*p* < 0.05). Under the intervention of sVAP, six categories were significantly affected (*p* < 0.05), with the Firmicutes phylum being a differential microbial group, while two dominant categories in the HFD group were affected (*p* < 0.05). These results suggest that sVAP-3K can regulate the gut microbial structure and function within the host.

### 2.9. LC-MS/MS Mass Spectrometry Detection

**Figure 12:**
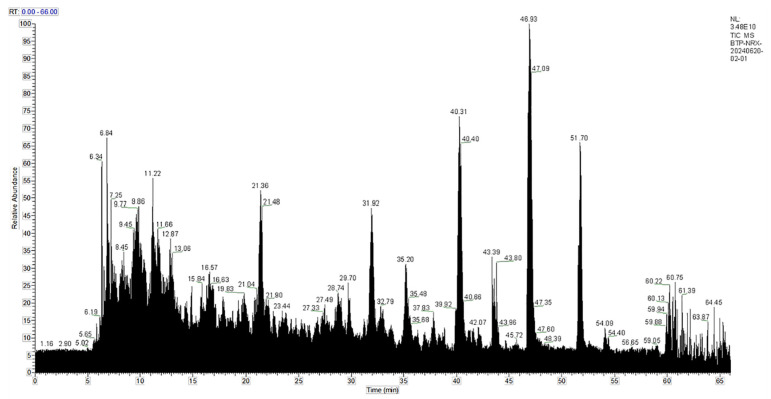
sVAP-3K component total ion chromatogram.

**Figure 13:**
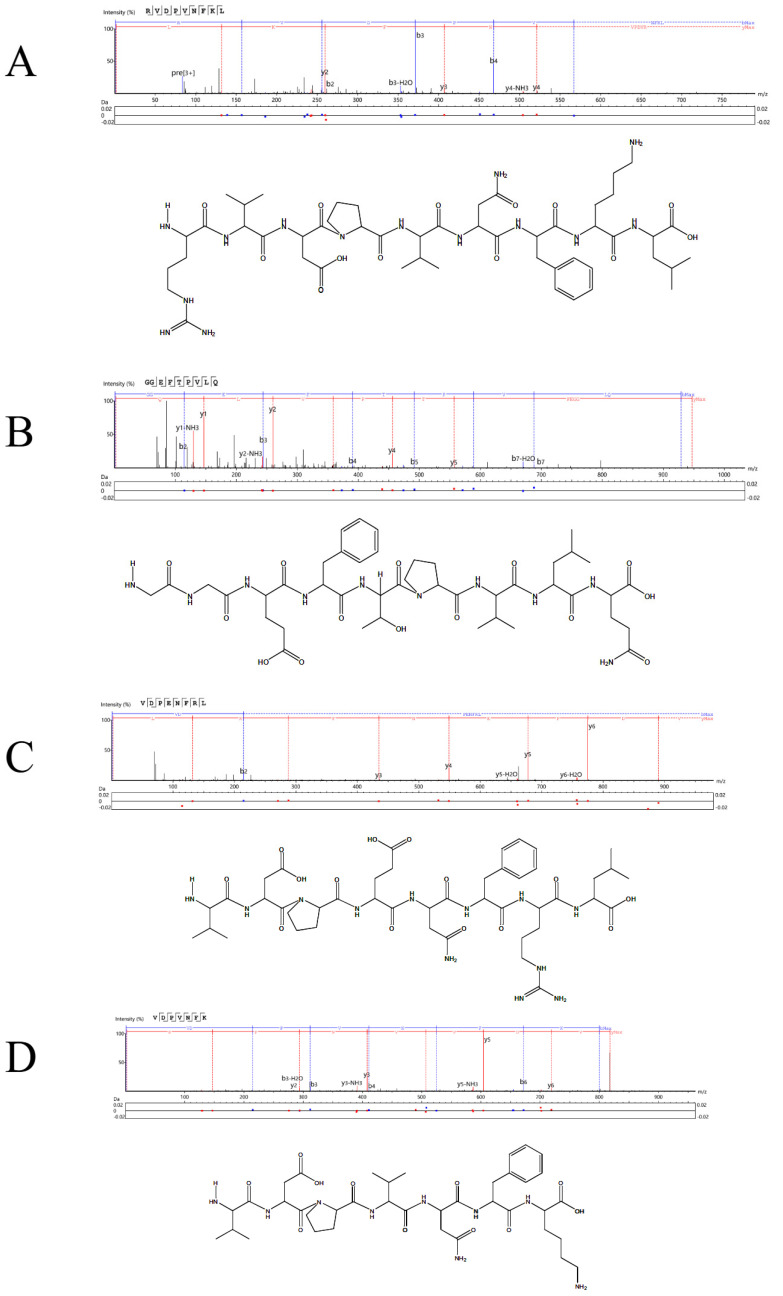
sVAP −3K secondary mass spectrometry image. (**A**) RVDPVNFKL; (**B**) GGEFTPVLQ; (**C**) VDPENFRL; (**D**) VDPVNFK.

The 3249 AA sequences were detected in sVAP-3K. The components separated by chromatography continuously enter the mass spectrum, and the mass spectrum is continuously scanned for data acquisition. A mass spectrum is obtained by scanning, and all ion intensities at different time points in the mass spectrum are added together to obtain a total ion current intensity. A Total Ion Chromatography (TIC) chart (MS1) with ion intensity as the y-axis and time as the x-axis was drawn, as shown in Figure 12. According to molecular docking, the sVAP-3K principal components (the top ten AA sequences with the highest content in primary mass spectrometry) were docked, and the top four AA sequences with the strongest binding energy were recorded to generate a secondary mass spectrum (i.e., MS2), as shown in Figure 13. The lowest energy value of RVDPVNFKL analyzed using Chemdraw 3D software 20.0 is −51.2065 kcal/mol, which contains six essential AAs and two non-essential Aas. The minimum energy value of GGEFTPVLQ is −26.7805 kcal/mol, which contains four essential AAs and four non-essential Aas. The minimum energy value of VDPENFRL is −53.6895 kcal/mol, which contains three essential AAs and three non-essential Aas. The minimum energy value of VDPVNFK is −16.1350 kcal/mol, which contains four essential AAs and two non-essential AAs.

### 2.10. Molecular Docking Results

**Figure 14:**
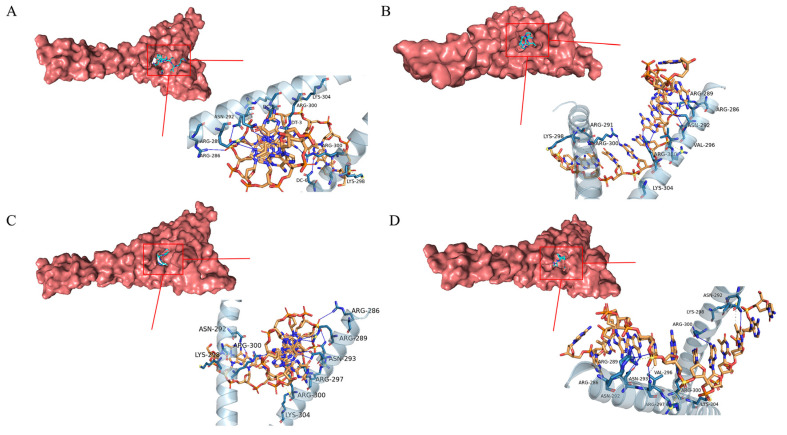
Docking results with 8K8C molecules. (**A**) RVDPVNFKL; (**B**) GGEFTPVLQ; (**C**) VDPENFRL; (**D**) VDPVNFK.

**Figure 15:**
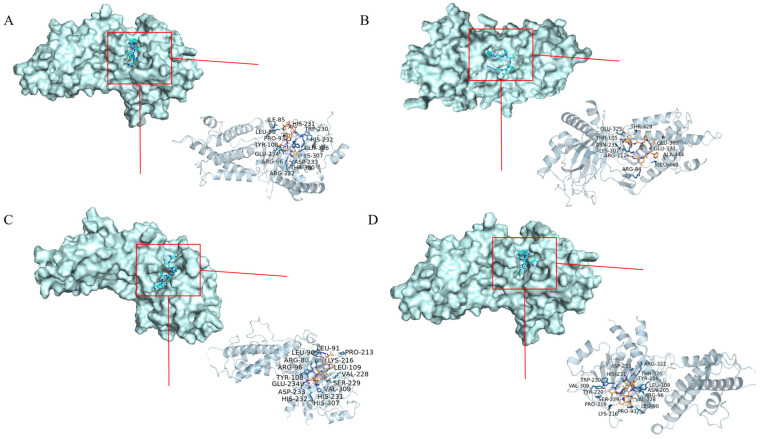
Docking results with 3LMF molecules. (**A**) RVDPVNFKL; (**B**) GGEFTPVLQ; (**C**) VDPENFRL; (**D**) VDPVNFK.

The top ten principal components with the most peak areas in the sVAP-3K mass spectrometry detection results were studied using molecular docking method. The AA sequences with binding potential in the sVAP-3K component were identified using 8K8C and 3LFM proteins, as shown in Table 1. When the binding affinity is less than −5 kcal/mol, the binding between the component and the target is moderate. When the binding affinity is less than −7 kcal/mol, the component target connection is strong [24], and the smaller the affinity, the stronger the binding. After screening, RVDPVNFKL, GGEFTPVLQ, VDPENFRL, and VDPVNFK had the highest Autodock Vina scores. Figure 14 and Figure 15 show the main target conformations of the above AA sequences. As shown in Figure 14, all four AA sequences can form 10 hydrogen bonds with residues in 8K8C and undergo two hydrophobic interactions. As shown in Figure 15, VDPENFRL forms 9 hydrophobic interactions, 17 hydrogen bonds, and 4 salt bridges with 3LFM; RVDPVNFKL forms 9 hydrophobic interactions and 15 hydrogen bonds with 3LFM; GGEFTPVLQ forms 8 hydrophobic interactions, and 8 hydrogen bonds with 3LFM; VDPVNFK forms 11 hydrophobic interactions, 10 hydrogen bonds, and 1 π–C bond with 3LFM. So, RVDPVNFKL, GGEFTPVLQ, VDPENFRL, and VDPVNFK may be potential active ingredients of sVAP-3K with therapeutic effects in obesity.

## 3. Discussion

Velvet antler is a typical traditional animal medicine with a history of over 2000 years of use. It has been used in traditional Chinese medicine to prevent and treat various human diseases [25,26]. However, studies on the anti-obesity effects of velvet antler are scarce. Therefore, based on previous research, this study evaluated the anti-obesity effect of sika deer velvet antler hydrolysates by induced–differentiated 3T3-L1 preadipocytes and HFD-induced C57BL/6 mouse models, and revealed the molecular mechanism of sVAP-3K. sVAP significantly inhibited lipid droplet formation in 3T3-L1 preadipocytes and reduced weight gain in obese mice fed with HFD.

While previous studies on velvet antler peptides have demonstrated their preventive effects on osteoporosis, liver injury, and their anti-inflammatory and antioxidant activities, information regarding their effects on adipocyte metabolism at the cellular level is limited. Therefore, this study investigated the effects of sVAP on 3T3-L1 adipocyte differentiation, initially screening for the optimal molecular weight fraction. In vivo verification tests were conducted on this optimal fraction, without further mechanistic investigation at the cellular level. We examined the ability of sVAP with different molecular weights to inhibit Lip-acc in 3T3-L1 adipocytes. Notably, sVAP exhibited a strong inhibitory effect on adipogenesis in differentiation-inducing media. Different molecular weight sVAP fractions demonstrated varying degrees of inhibition on Lip-acc during 3T3-L1 adipocyte differentiation, with sVAP-3K showing the strongest inhibitory effect on Lip-acc during the differentiation stage. Cytotoxicity assays were performed on seven different hydrolysates, determining a concentration of 50 μg/mL. Therefore, the Oil Red O staining experiment was conducted at this concentration, with the hydrolysates from neutral proteases displaying the best activity. After purifying three hydrolysates with different molecular weight ranges, cytotoxicity assays were again performed, determining a concentration of 200 μg/mL. Consequently, the Oil Red O experiment was carried out at this concentration, identifying the hydrolysates in the ≤3 kDa molecular weight range as having the best activity. According to previous experimental experience, the cytotoxic concentration of the purified samples should be lower than that of the crude extracts; however, the results of this experiment indicate that the cytotoxic concentration of the purified samples is higher than that of the crude extracts. The results suggest that all three purified different molecular weight samples have an inhibitory effect on adipocytes, with the ≤3 kDa samples showing the best effect, although none were more effective than the crude extract. The three different molecular weight samples may exhibit a synergistic effect, whereby their combined action on cells is more effective than their individual effects. Therefore, in vivo experiments were conducted to further verify the anti-obesity activity of sVAP-3K. Since adipocyte differentiation is accompanied by adipogenesis [27], we focused on the following signaling pathways to elucidate the anti-obesity mechanism of sVAP.

AMPK is a major cellular energy sensor and regulator of metabolic homeostasis, modulating protein, lipid, and glucose metabolism [28]. Activation of AMPK, through phosphorylation of Thr 172 in its α-subunit, redirects cellular metabolism [29]. The anti-obesity effects of sVAP may be associated with the activation of the AMPK signaling pathway, thereby enhancing energy metabolism. AMPK activation, as a key upstream regulator, inhibits adipogenesis by downregulating C/EBPα [30]. Adipocyte differentiation is regulated by multiple signaling pathways and transcription factors. C/EBPα are key transcription factors regulating numerous transcriptional pathways involved in adipocyte differentiation and adipogenesis [31,32]. During adipocyte differentiation, C/EBPα play crucial roles in a complex transcriptional cascade [33], mutually stimulating their expression and cooperatively activating many metabolic adipocyte genes [34].

Data showed no significant difference in weekly food intake between the sVAP-3K, CON, and HFD groups, but a significant difference in body weight was observed. In conjunction with AMPK signaling pathway analysis, our findings indicate that sVAP-3K exerts its anti-obesity effect not by suppressing appetite and reducing food intake, but rather by modulating energy metabolism. In this study, we determined that sVAP-3K exerts its anti-obesity effects both in vitro and in vivo by activating AMPK to inhibit adipogenesis.

Concurrently, we examined the levels of various biochemical indicators in the serum of HFD mice in each group. Compared to the normal control group, mice in the HFD group exhibited significantly higher serum total cholesterol and triglyceride levels. Administration of sVAP-3K to HFD-fed mice at doses of 150 mg/kg and 300 mg/kg showed a significant, dose-dependent decrease in body weight, fat weight, and serum triglyceride levels, without a significant change in food intake. Obesity is associated with the development of various metabolic disease conditions, such as type 2 diabetes and non-alcoholic fatty liver disease [35]. Based on GOT and GPT data, the HFD group showed some degree of liver damage, but this was significantly reduced in a dose-dependent manner in the sVAP-3K-treated groups.

The gut and liver are directly connected through the portal vein, influencing liver lipid metabolism Via the “gut-liver axis” [36]. The human gut hosts approximately 10^14^ bacteria, which regulate the digestion and absorption of ingested food, playing a crucial role in determining the conversion rate of ingested energy. The four main phyla in the gut are Firmicutes, Bacteroides, Proteobacteria, and Actinobacteria, accounting for 90% of the bacterial composition of the gut microbiota [37]. In recent years, increasing evidence suggests that obesity is linked to gut microbiota, making gut microbiota management a new approach in obesity treatment [38]. The results of this study show that sVAP can improve the gut microbiota structure in HFD-fed mice, bringing it closer to the ecological state of the gut microbiota in normal mice. However, obese individuals and animals exhibit a higher Firmicutes/Bacteroidetes (F/B) ratio compared to those with normal weight, making this ratio a marker for obesity [39]. Our study confirms that a high-fat diet leads to an increased F/B ratio in the gut microbiota of mice, while in the sVAP group, this ratio decreases, indicating that sVAP has the effect of regulating and ameliorating gut microbiota dysbiosis. Research has shown that Desulfovibrionales produce H_2_S in the colon, which inhibits colonocyte respiration and the production of short-chain fatty acids like butyrate [40,41]. Our study indicates an increased relative abundance of Desulfovibrionales in the gut microbiota of HFD mice, while this abundance decreases in the sVAP group.

Almost all HFDs promote weight gain and lead to hepatic steatosis [42]. The liver coordinates AA metabolism and controls systemic AA exposure [43]. AA derivatives have shown beneficial effects in promoting weight loss and improving lipid profiles, primarily through mechanisms including inhibiting lipogenesis, increasing lipolysis, promoting adipogenesis, and improving glucose metabolism [44]. sVAP-3K contains 3249 AA sequences, including 3042 oligopeptides (composed of 2–20 AAs) and 207 polypeptides (composed of more than 20 AAs). AA sequences without reference value were discarded based on ppm data, resulting in 3016 effective AA sequences (203 polypeptides and 2813 oligopeptides). The top ten most abundant AA sequences were selected for molecular docking, all of which were oligopeptides ranging from 2 to 10 AAs in length. Docking with 8K8C and 3LFM revealed four sequences with strong binding affinity: RVDPVNFKL, GGEFTPVLQ, VDPENFRL, and VDPVNFK. These four AA sequences were not found in the NCBI database, indicating that they are novel anti-obesity amino acid sequences isolated for the first time from Cervus nippon velvet antler.

Studies have demonstrated that cysteine deficiency promotes rapid and significant AMPK activation [45]. Furthermore, the potential effects of trigonelline, linoleic acid, glutamine, and L-aspartic acid on obesity treatment or pathogenesis have been reported in other representative metabolites [46,47,48,49]. Consistent with our findings, LC-MS/MS analysis combined with molecular docking revealed that four AA sequences within the major components of sVAP-3K exhibit strong anti-obesity activity. These four sequences contain AA previously shown to be effective in obesity treatment. Therefore, the most potent anti-obesity AA sequences within sVAP-3K hold promise for translation into natural products to address the obesity challenge. The four identified AA sequences all contain valine (Val), proline (Pro), and phenylalanine (Phe). Branched-chain AAs, including Val and leucine (Leu), have been reported to mediate anti-obesity effects, particularly in rodent models, where BCAA diets promote lean body mass in obesity or metabolic dysfunction, or increase satiety leading to weight loss [50]. Tryptophan (Trp), histidine (His), and Phe metabolites, in their respective metabolic pathways, can influence obesity and diabetes [51]. Phe complexes sensitize insulin signaling pathways by activating AMPK (AMP-activated protein kinase) phosphorylation [52], enhancing insulin sensitivity by amplifying insulin-induced Akt phosphorylation and increasing insulin-stimulated glucose uptake in adipocytes [53]. Based on the inhibitory effects of various AAs on obesity and in vivo and in vitro activity experiments, we conclude that the naturally occurring oligopeptides isolated from velvet antler of sika deer indeed possess anti-obesity effects, providing foundational data for the development of natural and safe anti-obesity drugs.

## 4. Materials and Methods

### 4.1. Drugs and Materials

We obtained the first two branched velvet antler from the Northeast subspecies of Sika deer (*Cervus nippon* Temminck) at five years old from Jilin Hongyu Deer Industry Technology Co, Ltd., Shuangyang District, Changchun City, Jilin Province, China, which was identified as Sika deer velvet antler by Professor Lin Zhe from the School of Pharmaceutical Sciences, Changchun University of Chinese Medicine. Mouse 3T3-L1 preadipocytes were obtained from the American Type Culture Collection (ATCC, Rockville, MD, USA). We used 30 Dulbecco modified Eagle medium (DMEM) (PM150210), fetal bovine serum (FBS) (164210), neonatal bovine serum (NBS), penicillin streptomycin (PS), and phosphate-buffered saline (PBS) (Wuhan Prichella Biotechnology Co., Ltd., Wuhan, China) for cell culture. Pepsin (P8160), trypsin (9002-07-7) (Solarbio, Beijing, China), chymotrypsin (9004-07-3), dispase (9068-59-1), alcalase (9014-01-1) (Shanghai McLean Biochemical Technology Co., Ltd., Shanghai, China), and protamex (S10155-5g) (Shanghai Yuanye Biotechnology Co., Ltd., Shanghai, China) were used for enzymatic hydrolysis of sika deer velvet antler. Orlistat (Hangzhou Zhongmei Huadong Pharmaceutical Co., Ltd., Hangzhou, China H20100190) is used as the positive drug. Isopropanol (Analytical level, 99.7%, S005280, Wuhan, China), 3- (4, 5-dimethylthiazol-2-yl) 2, 5-diphenyltetrazolium bromide (298-93-1), 3-isobutyl-1-methylxanthine (No. I8450), dexamethasone (DEXA) (50-02-2), insulin (S114196), and Oil Red O (1320-06-5) (Solarbio, Beijing, China) were used for cell induction. Phospho-AMPK (Thr172) (AA393), CEBP alpha Rabbit Monoclonal Antibody (AF2590), PPARγ Rabbit Polyclonal Antibody (AF7797) (Shanghai Biyuntian Biotechnology Co., Ltd., Shanghai, China), enzyme-linked immunosorbent assay (ELISA) kit for high-density lipoprotein cholesterol (JT-004729), low-density lipoprotein cholesterol (JT-004730), total cholesterol (JT-004731), triglycerides (JT-004732), aspartate aminotransferase (JT-004733), and alanine aminotransferase (JT-004734) (Solarbio, Beijing, China); AMPK Alpha Polyclonal Antibody (10929-2-AP), GADPH (10494-1-AP), and Rabbit IgG (30000-0-AP) (Proteintech Group, Manchester, UK) were all used as biochemical indicators for mouse experiments.

### 4.2. Preparation of Sample

#### 4.2.1. Preparation of Velvet Antler Hydrolysate

The velvet antler was sliced, freeze-dried, and then ground into a powder. Velvet antler coarse powder was homogenized with disposable distilled water (1:20 g/mL), and then pepsin, trypsin, chymotrypsin, multi-enzyme, dispase, alcalase, and protamex were added at a 1:100 enzyme base ratio: extraction conditions are shown in Table 2. The mixture was then placed in a shaker for enzymatic hydrolysis for 24 h, the hydrolysate was then boiled at 100 °C for 15 min to inactivate the enzyme. It was then allowed to sit at room temperature, and the pH was adjusted to 7.0. After centrifuging at 4000× *g* for 20 min, any unhydrolyzed residue from the supernatant hydrolysate was removed [54]. The resultant hydrolysate solution was lyophilized and stored at −20 °C for further use.

#### 4.2.2. Preparation of Hydrolysate with Different Molecular Weights

The ultrafiltration device Tanfil 100 (Rocker, Taiwan, China) was used, equipped with an Omega membrane to separate sVAP into samples with molecular weights of ≤3 KDa, 3–10 KDa, and ≥10 KDa. The resultant hydrolysate with different molecular weights was lyophilized and stored at −20 °C for further use.

### 4.3. In Vitro Experiments

#### 4.3.1. Cell Culture and Cell Differentiation

3T3-L1 preadipocytes were cultured at 37 °C and 5% CO_2_ within DMEM containing 10% BS and 5% PS until they reached more than 80% confluence. Fully fused 3T3-L1 preadipocytes were induced in differentiation induction medium I and II according to reference [55], but some concentrations were modified in this experiment (2.5 mM DEXA, 800 μM insulin, and 2N NaOH). 3T3-L1 preadipocytes cells were differentiated into adipocytes after 2 days, cultured with differentiation induction medium II, and replaced every 2 days until day 8. The cells had fully differentiated into mature adipocytes.

#### 4.3.2. Cytotoxicity Assay

3T3-L1 adipocyte viability was assessed using the MTT assay. The previous text described the cell differentiation and treatment with sVAP (with and without sVAP, 25 μL per well). The concentrations of the seven different hydrolysates are 50 μg/mL; the concentrations of the three different molecular weight hydrolysates are 12.5, 25, 50, 100, and 200 μg/mL. The cells were cultured in a CO_2_ incubator for 24 h, and 100 μL of MTT solution (2 mg/mL in PBS) was added to each well [54]. After 3 h of incubation, the supernatant was discarded, and 300 μL of DMSO was added to each well for dissolution. After shaking in the dark for 10 min, the absorbance value of each well was measured on an ELISA plated reader at a wavelength of 540 nm, which can indirectly reflect the number of live cells.

#### 4.3.3. Oil Red O Staining

Lipid accumulation of differentiated 3T3-L1 adipocytes was determined by Oil Red O staining. 3t3-l1 preadipocyte cells were seeded into 12-well plates and maintained for two days after reaching confluence. The concentrations of the seven different hydrolysates are 50 μg/mL; the concentration of the three different molecular weight hydrolysates is 200 μg/mL. Cultural media were replaced with differentiation medium, and the medium was changed every 2 days. Cells were treated with sVAP at day 0, 2, 4, and 6 of adipogenesis. On day 8, cells were stained with Oil Red O. Briefly, cells were washed with PBS and fixed in 10% Formalin for 60 min, washed with 10% isopropanol and air-dried. Cells were then stained with ORO staining (0.5 g in 100 mL isopropanol) for 1 h at room temperature. After removing the staining solution, following multiple water washes, they were air-dried. Lipid droplets were observed with a microscope (20×), then eluted with 100% isopropanol and quantified by measuring the absorbance at 540 nm using a microplate reader [56].

### 4.4. In Vivo Experiments

#### 4.4.1. Animals

SPF grade male Kunming (C57BL/6) mice (3–4 weeks old, weight 18–20 g) were purchased from Liaoning Changsheng Biotechnology Co., Ltd., Liaoning, China (animal license number: SYXK (Liao) 2020-0001). The maintenance feed for mice was purchased from Beijing Ke’ao Xieli Feed Co., Ltd., Beijing, China. Healthy male C57BL/6 mice were maintained in a temperature-controlled room (22 ± 1 °C) with a 12 h light/dark cycle and free access to normal food and water. The animals were allowed to adapt to the environment for one week before starting the experiment. All animal experimental protocols used in this study were approved by the Institutional Animal Care and Use Committee of Changchun University of Chinese Medicine. The approval number for IACUC is 2024520.

#### 4.4.2. HFD-Induced Obesity Animal Model

An obese mouse model was created using a high-fat diet (Table 3) for 4 weeks. Mice gaining >20% body weight were divided into 5 groups (*n* = 8/group): control (normal diet, water gavage), HFD (high-fat diet), HFD-P (orlistat, 10 mg/kg) [1], HFD-L (sample, 150 mg/kg), and HFD-H (sample, 300 mg/kg) [18]. Treatment lasted 11 weeks. Weekly body weight and food intake were recorded. A multimodal in vivo imaging system (In Vivo Xtreme) was used to perform CT imaging of mice in each group to confirm the composition of body fat in each group. Abdominal cavity injection of 1% pentobarbital sodium (50–90 mg/kg) was used for anesthesia. Blood was collected from the mouse’s eyeball. Serum was collected by centrifugation at 4 °C and then stored at −80 °C. Anesthetized mice were euthanized by cervical dislocation; adipose and liver tissues were collected and stored at −80 °C.

#### 4.4.3. Analysis of Sugar Tolerance and Oral Glucose Tolerance Test

Mice were fasted overnight prior to euthanasia, and fasting blood glucose levels were measured using a glucometer (Haier, Qingdao, China). The blood glucose level of mice after overnight fasting was measured (0 min). In the oral glucose tolerance test (OGTT), the mice were orally administered with 20% glucose solution (0.2 mL/20 g). Blood samples were collected from the tail vein and monitored at 15, 30, 60, and 120 min, and the area under the curve (AUC) was calculated.

#### 4.4.4. Enzyme-Linked Immunosorbent Assay (ELISA)/Serum Chemistry Analysis

All mice were fasted for 12 h before sacrifice. Blood was collected and separated via by centrifugation at 3000× *g*, and 4 °C for 15min. The HDL-H, HDL-L, TC, TG, AST, and ALT levels in the serum were measured using commercial ELISA kits according to the manufacturer’s instructions.

#### 4.4.5. Western Blot Analysis

Mouse liver and adipose tissue (30 mg) lysates were prepared, protein concentrations were subsequently determined using an Enhanced BCA Protein Assay Kit (Shanghai Yuanye Biotechnology Co., Ltd., Shanghai, China). A measure of 50 μg protein/sample was separated by 10%SDS-PAGE, transferred to PVDF membrane (Merck Millipore, Darmstadt, Germany). The primary antibodies used were as follows: P-AMPK (1:1000), AMPK (1:1000), C/EBPα (1:1000) PPARγ (1:500), and GAPDH (1:1000). After washing with 1×TBST containing 0.1% (*v*/*v*) Tween-20, it was incubated with the secondary antibody IgG. The grayscale of protein bands was quantified by the automatic chemiluminescence imaging system (Gelview 6000Plus, Guangzhou Biolight Biotechnology Co., Ltd., Guangzhou, China). Relative densitometry was measured with Image J software (Java 1.8.0_345; https://imagej.nih.gov/ij).

#### 4.4.6. Histomorphological Analysis

After obtaining abdominal fat tissue and liver tissue, fix them overnight in 4% paraformaldehyde [1]. Slice the paraffin embedded abdominal fat or liver and stain with hematoxylin, eosin, and ORO. Observe the stained sections separately using a slice scanning microscope (Shenzhen Shengqiang Technology Co., Ltd., Shenzhen, China, SR-S500) and an upright optical microscope (Olympus CX31, Tokyo, Japan), and analyze the ORO section values using Image J software.

#### 4.4.7. Gut Microbiota Analysis

The cecal contents were collected, immediately frozen in liquid nitrogen for DNA extraction, and stored at −80 °C until use. Total DNA from microbial communities in mouse cecal contents was extracted following the instructions of the MagAttract PowerSoil Pro DNA Kit (Qiagen, Hilden, Germany). The quality of the extracted DNA was assessed using 1% (*w*/*v*) agarose gel electrophoresis. DNA concentration and purity were measured with a NanoDrop 2000. The V3-V4 region of the 16S rRNA gene was amplified by PCR using primers 338 F (5′ACT CCT ACG GGA GGC AGC AG-3′) and 806 R (5′-GGA CTA CHVGGG TWT CTAAT-3′) [57]. The PCR products from pooled samples were checked on a 2% (*w*/*v*) agarose gel. The amplified products were purified using the AxyPrep DNA Gel Extraction Kit (Axygen Biosciences, Union City, CA, USA) and verified by 2% (*w*/*v*) agarose gel electrophoresis. The recovered products were quantified using a Quantus™ Fluorometer (Promega, San Luis Obispo, CA, USA). A database was constructed using the NEXTFLEX^®^ Rapid DNA-Seq Kit. Sequencing was performed on the Illumina NextSeq 2000 PE300 platform.

Using the Uparse software v7.0.1001 platform, species richness, diversity, and composition were analyzed with indices such as Ace, Chao, Coverage, Shannon, Simpson, Sobs, and Venn diagrams. At the phylum level, the community composition and the relationships between samples and species within groups were analyzed using R language (vegan). Hierarchical clustering based on a distance matrix (hcluster) was performed, followed by principal coordinate analysis using vegan. LEFSe employed Linear Discriminant Analysis (LDA) to estimate the impact of species abundance on differential effects and to identify species with significant differences between groups. The effect size is 4.

### 4.5. Structural Identification

#### 4.5.1. LC-MS/MS Mass Spectrometry Detection

A 1 mg sample was reduced (DTT), alkylated (IAM), and desalted [58]. The concentration was determined Via ELISA (480 nm). LC-MS/MS analysis (Easy nLC 1200/QExactive) used a C18 column (gradient elution) for peptide separation. MS1 (70, 000 resolution) identified the top 10 peptides; MS2 (17, 500 resolution, 28 eV collision energy) characterized the most active peptide. Raw MS data were generated.

#### 4.5.2. Molecular Docking

Molecular docking is one of the most commonly used methods because it can predict the conformation of small molecule ligands within appropriate target binding sites with considerable accuracy. Use Chemdraw 3D to draw the amino acid sequence and perform energy minimization. Using Autodock Version 1.5.2 software for molecular docking, protein ligand interactions were characterized by the number of contacts between residue atom pairs at different distance intervals. 8K8C (Crystal structure of C/EBPalpha BZIP domain bound to a high affinity DNA) and 3LFM (Crystal structure of the fat mass and obesity associated (FTO) protein reveals basics for its substrate specificity) were selected as receptor proteins, and the top ten AA sequences of the peak area were detected by LC-MS/MS mass spectrometry as ligands. Using PyMOL(TM) Molecular Graphics System, Version 2.1.0. to analyze the anti-obesity activity of high binding energy AA sequences and predict the optimal AA sequence.

### 4.6. Statistical Analysis

Graphpad Prism 10.1.2 software was used to plot and compare statistically significant values, and multiple comparisons were evaluated using one-way analysis of variance (ANOVA). Assays were carried out in triplicate, and results were expressed as mean values ± standard deviation (SD), and *p* < 0.05 indicates statistical significance.

## 5. Conclusions

sVAP can ameliorate obesity by inhibiting adipogenesis, regulating metabolism in the body, expenditure, and improving gut microbiota dysbiosis. This provides evidence for its potential application in the prevention and treatment of obesity, offering great potential as a novel drug for obesity prevention and treatment.

## Figures and Tables

**Table 1:** Binding affinity results of 8K8C and 3LFM with ligands (kcal/mol).

Ligand	Length	m/z	Area (×10^8^)	Receptor PDB ID	Binding Affinity (ΔG in kcal/mol)	Receptor PDB ID	Binding Affinity (ΔG in kcal/mol)
TT	2	221.11111	3.25	3LFM	−6.5	8K8C	−6.3
TVKKI	5	588.40930	1.26		−6.1		−7.0
TVKKL	5	588.40930	1.26		−6.0		−7.1
DPVNFKL	7	416.73251	1.36		−7.0		−8.5
VDPVNFK ^a^	7	818.44043	1.48		−7.7		−8.5
VDPENFRL ^a^	8	495.25735	1.89		−8.7		−8.6
VDPVNFKL	8	931.52509	4.75		−7.9		−8.1
RVDPVNFKL ^a^	9	363.21371	1.36		−7.7		−8.9
GGEFTPVLQ ^a^	9	474.24643	1.03		−7.6		−8.7
SDLSDLHAHK	10	374.85684	7.96		−8.0		−8.1

^a^ To screen the AA sequence with the strongest binding energy and the best anti-obesity activity by combining the two results.

**Table 2:** Extraction conditions of different proteases.

Protease	Temperature (°C)	pH
Pepsin	37	2.0
Trypsin	37	7.6
Chymotrypsin	37	7.8
Multi-enzyme (Pesin:Trysin:Chymotrypsin = 1:1:1)	37	7.0
Dispase	50	6.0
Alcalase	50	8.0
Protamex	50	6.0

**Table 3:** Ingredient composition of a normal and high-fat diet.

Nutritional Components	Normal Diet (%)	High Fat Diet (%)
Crude protein	≥20.0	≥15.0
Crude fat	≥4.0	≥12.0
Coarse fiber	≥8.0	≤5.0
Crude ash content	≥9.0	≤8.0
Water content	≤8.0	≤10.0
Ca	1.0–2.0	0.8–1.6
Total phosphorus	0.4–0.8	0.5–1.0
Energy content	2.480 kcal/g	3.941 kcal/g

## Data Availability

Data will be made available on request.

## Supplementary Materials

The following supporting information can be downloaded at: https://www.mdpi.com/article/10.3390/molecules30051173/s1, Figure S1: Liver P-AMPK; Figure S2: Liver AMPK; Figure S3: Liver C/EBPα; Figure S4: Liver PPARγ; Figure S5: Fat P-AMPK; Figure S6: Fat AMPK; Figure S7: Fat C/EBPα; Figure S8: Fat PPARγ; Figure S9: GADPH.

## Abbreviations

AA	Amino acid
AMPK	The AMP activated protein kinase
AUC	Area under the curve
C/EBPα	CCAAT/enhancer binding protein–α
DH	Dispase hydrolysates
DMEM	30 Dulbecco modified Eagle medium
DEXA	Dexamethasone
ELISA	Enzyme-linked immunosorbent assay
FBS	Fetal bovine serum
HFD	High-fat diet
Lip-acc	Lipid accumulation
NBS	Neonatal bovine serum
ORO	Oil Red O
OGTT	Oral glucose tolerance test
PS	Penicillin streptomycin
PBS	Phosphate-buffered saline
Phe	Phenylalanine
sVAP	Sika deer velvet antler peptides
sVAP-3K	Sika deer velvet antler peptides ≤ 3 KDa
WAT	White adipose tissue
WHO	World Health Organization
