# Supplementary figures and images for: The Effects of Sika Deer Antler Peptides on 3T3-L1 Preadipocytes and C57BL/6 Mice via Activating AMPK Signaling and Gut Microbiota
Source: Molecules. 2025 Mar 6;30(5):1173. doi: 10.3390/molecules30051173 (PMC11901460; doi:10.3390/molecules30051173)

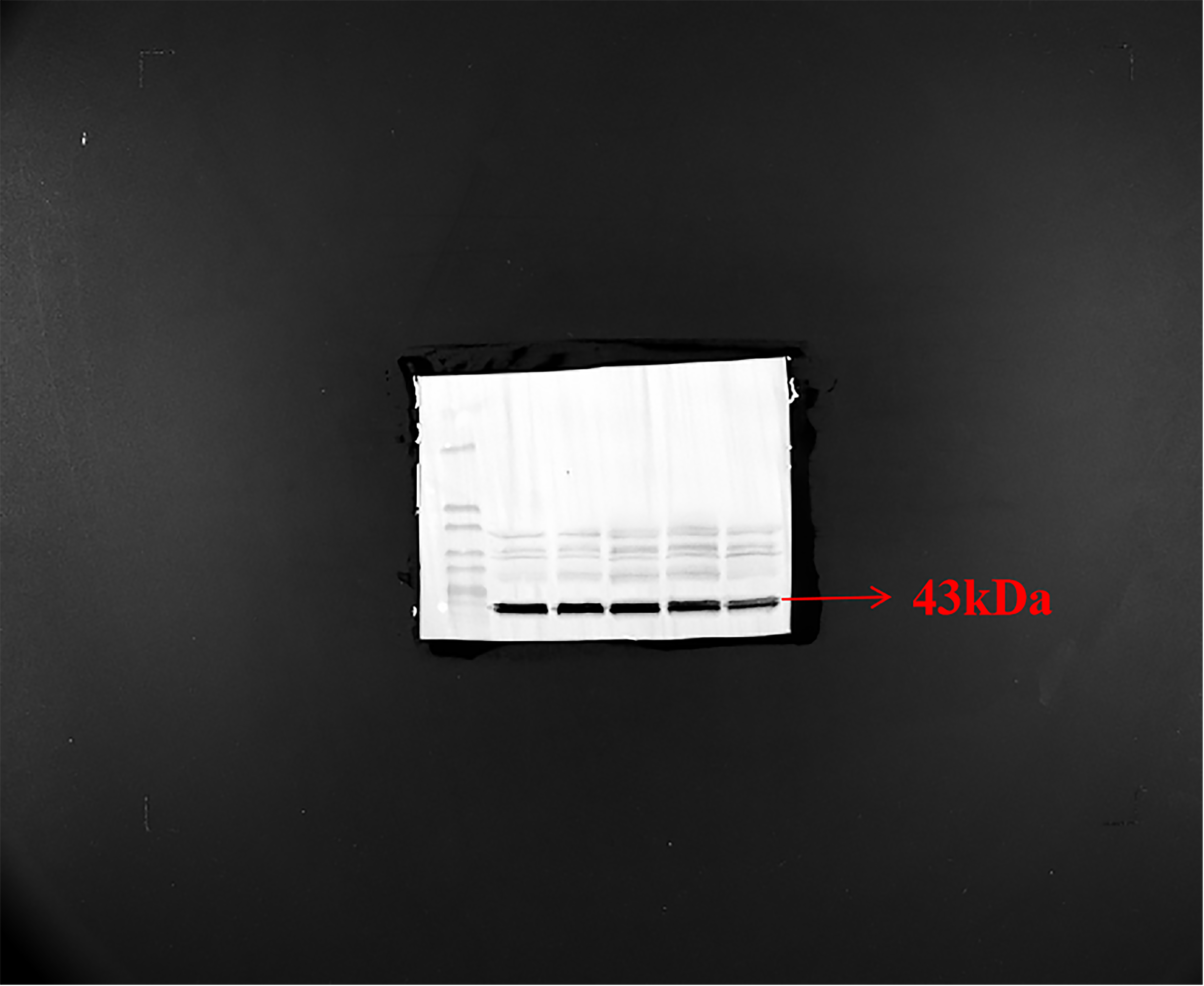

Supplement: Supplementary file 1 [file molecules-30-01173-s001.zip › Figure S3 Liver CEBP.tif]

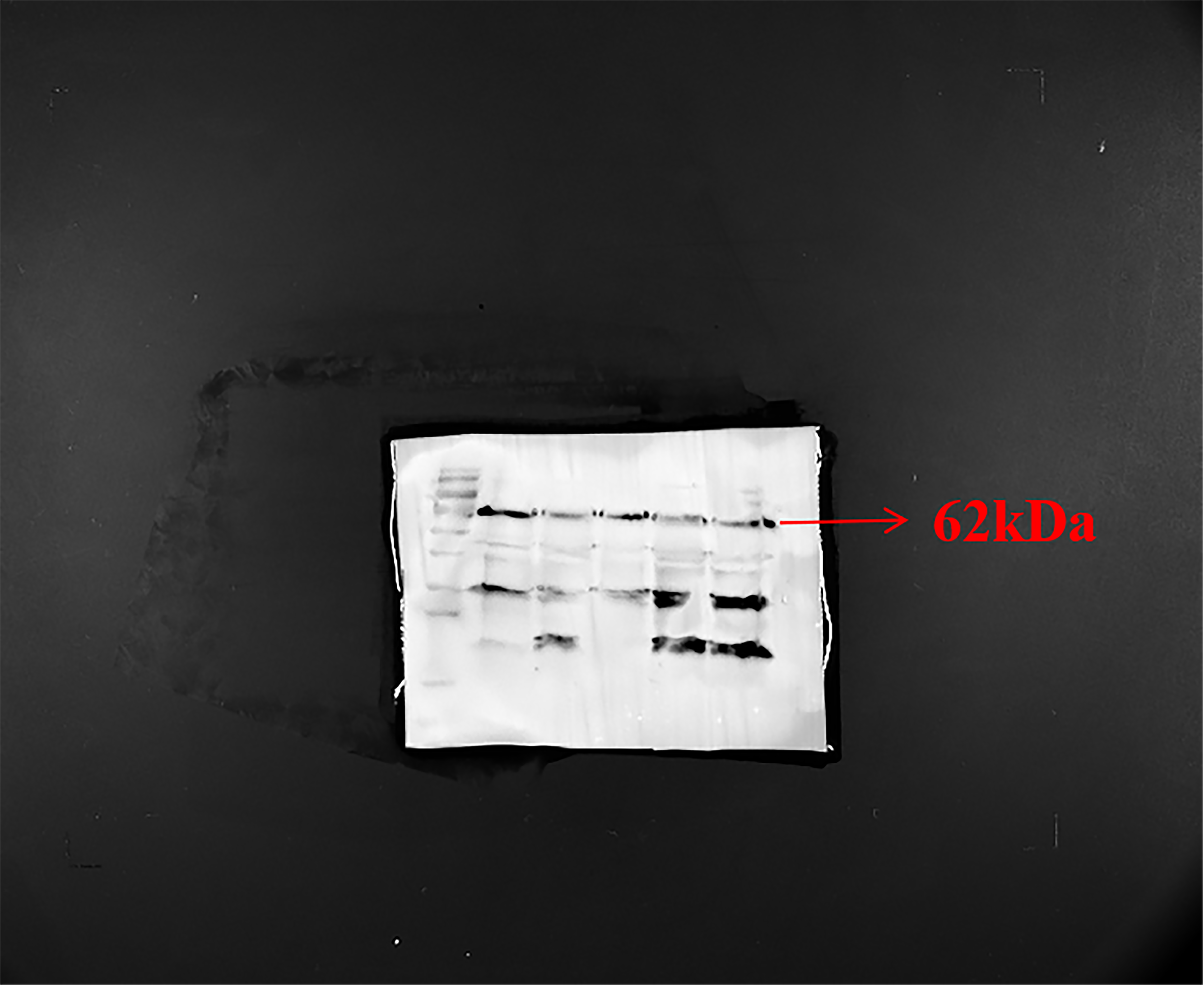

Supplement: Supplementary file 1 [file molecules-30-01173-s001.zip › Figure S4 Fat P-AMPK.tif]

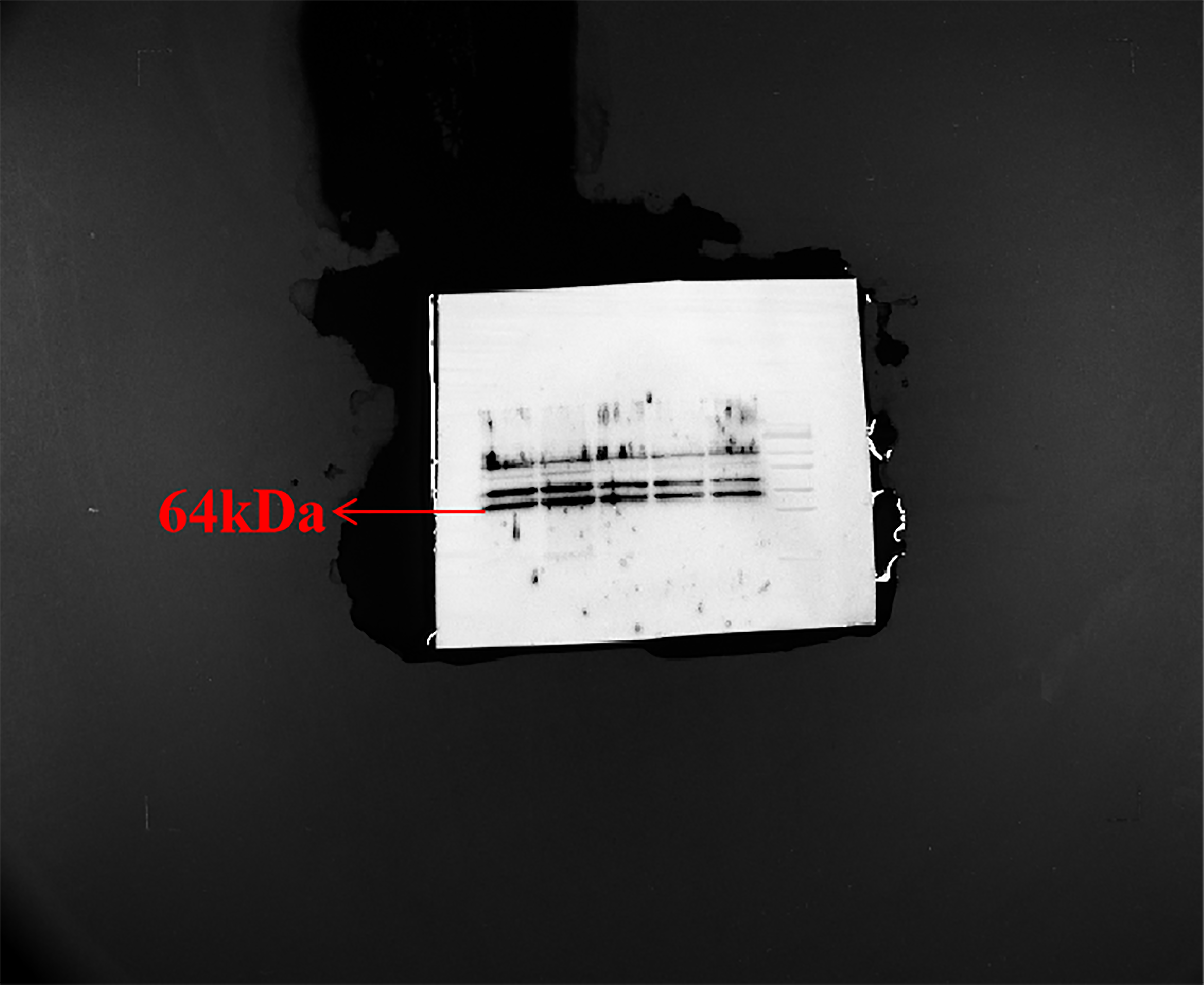

Supplement: Supplementary file 1 [file molecules-30-01173-s001.zip › Figure S5 Fat AMPK.tif]

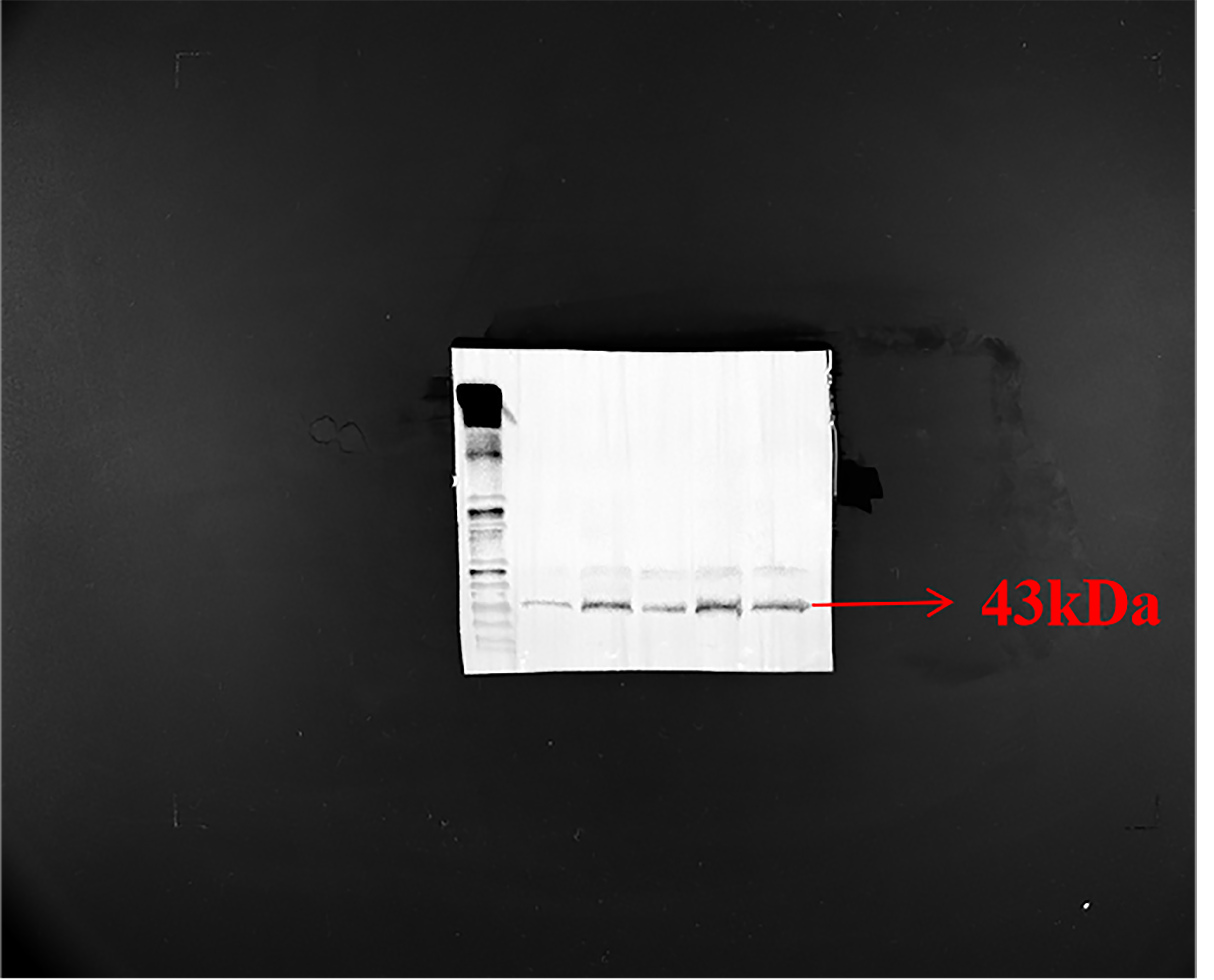

Supplement: Supplementary file 1 [file molecules-30-01173-s001.zip › Figure S6 Fat CEBP.tif]

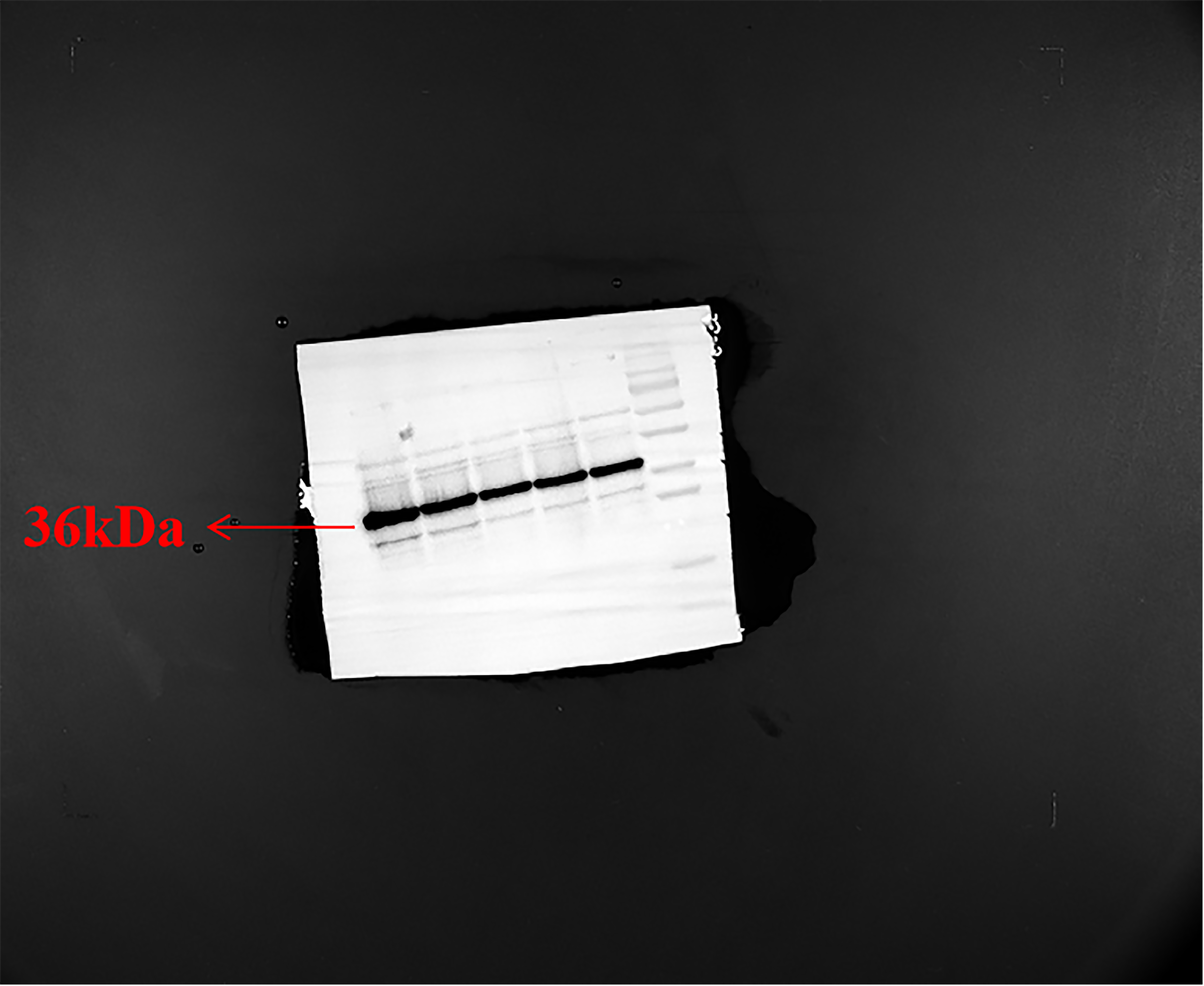

Supplement: Supplementary file 1 [file molecules-30-01173-s001.zip › Figure S7 GADPH.tif]

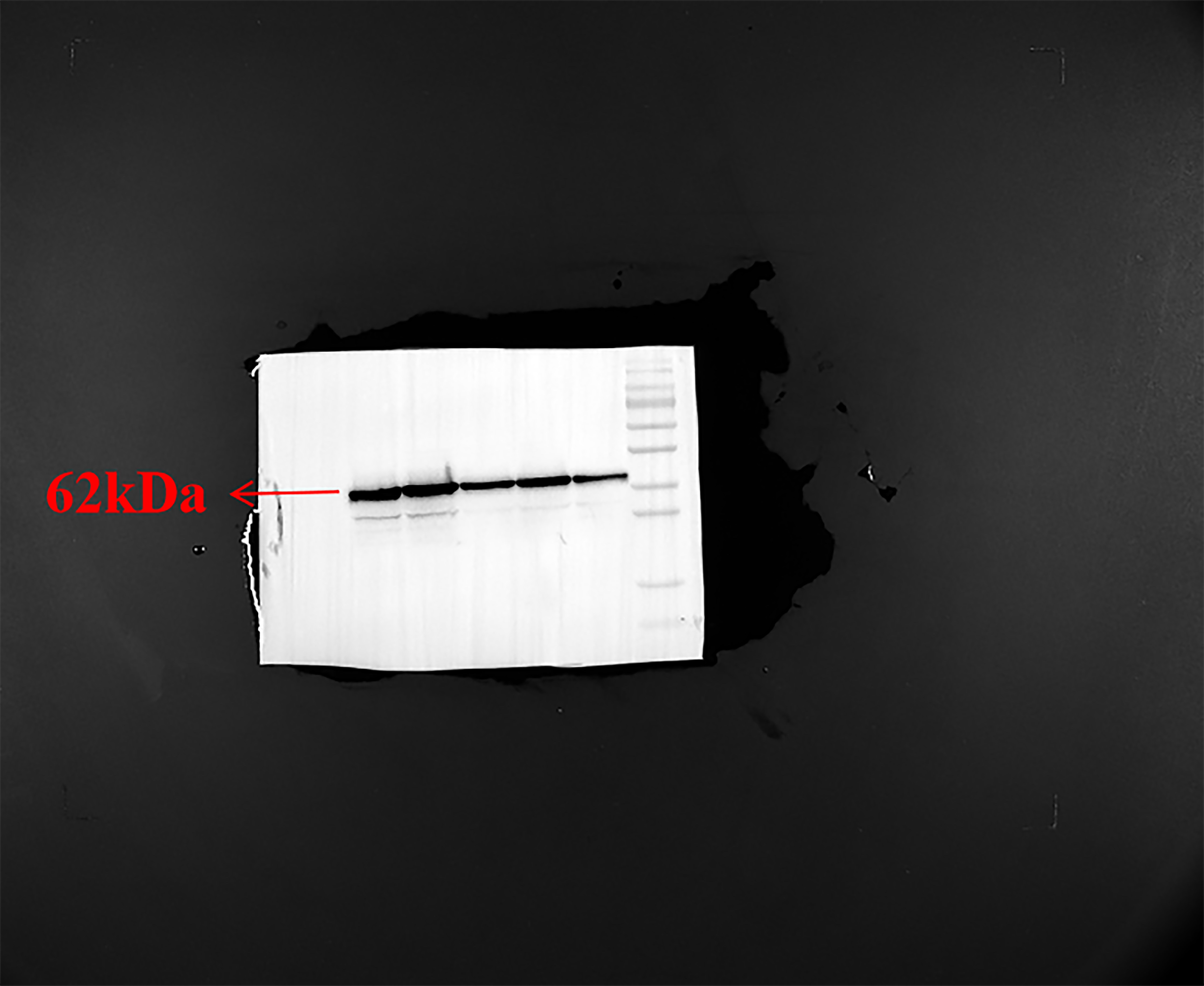

Supplement: Supplementary file 1 [file molecules-30-01173-s001.zip › Figure S1 Liver P-AMPK.tif]

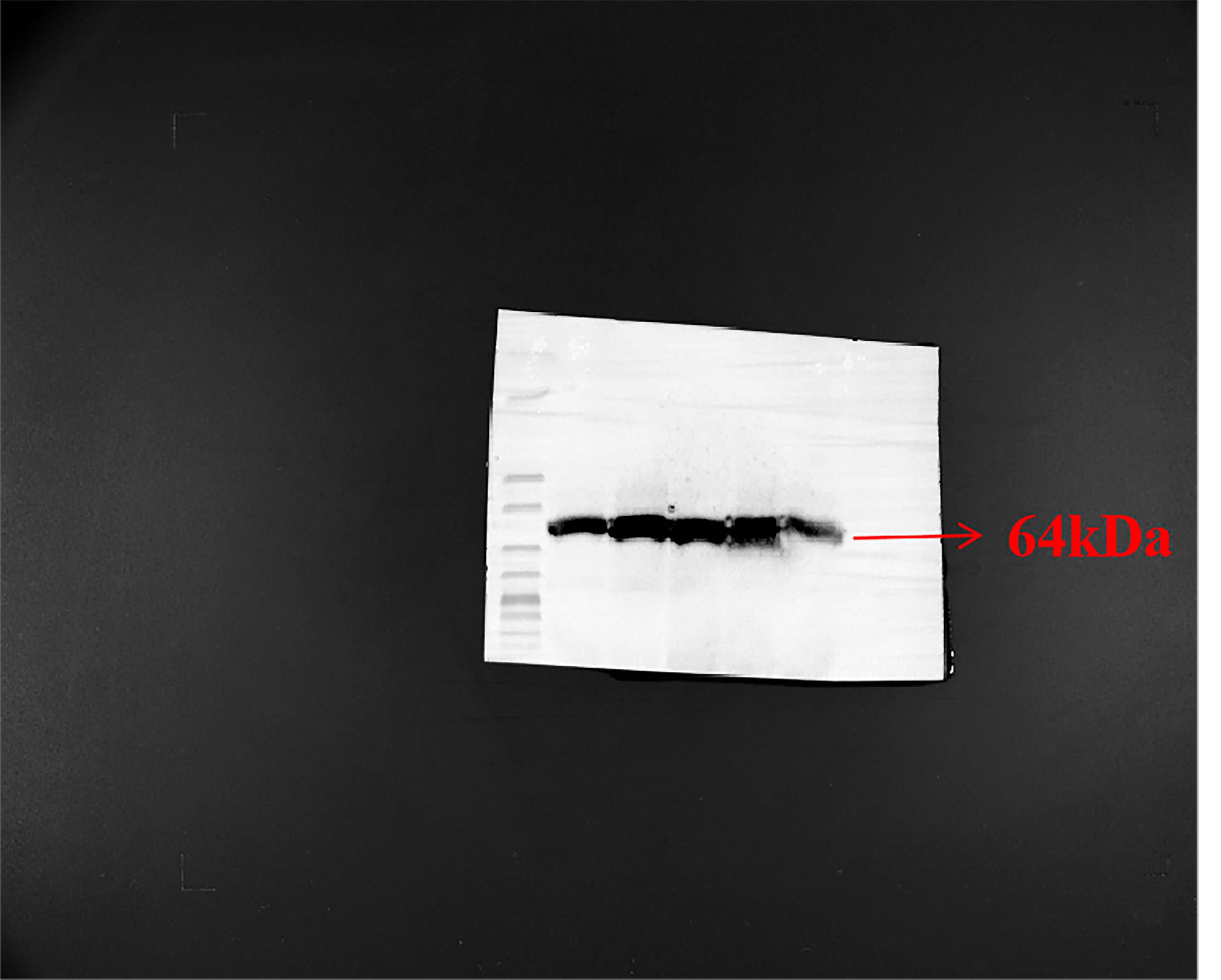

Supplement: Supplementary file 1 [file molecules-30-01173-s001.zip › Figure S2 Liver AMPK.tif]

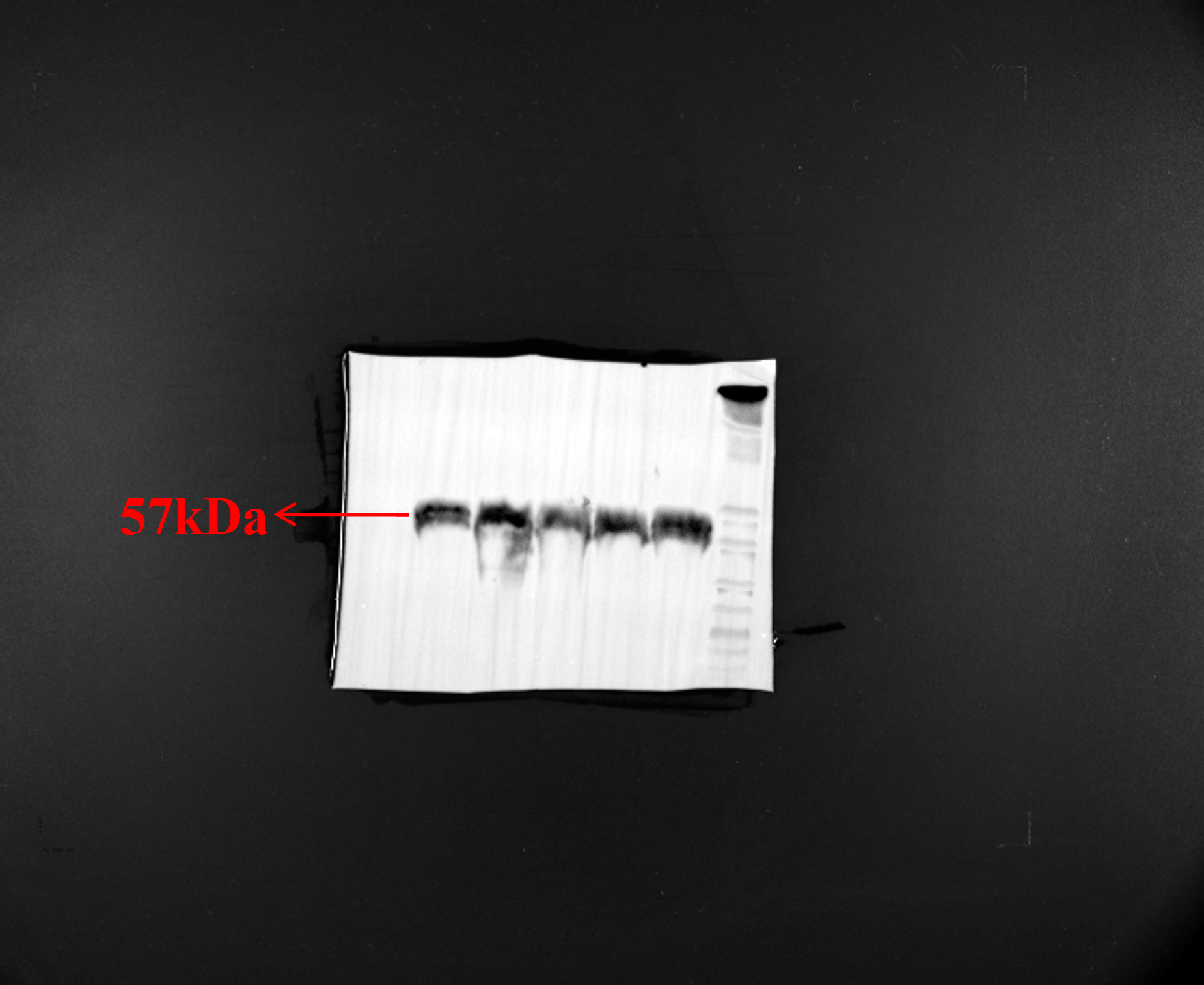

Supplement: Supplementary file 1 [file molecules-30-01173-s001.zip › Figure S4 Liver PPARγ.tif]

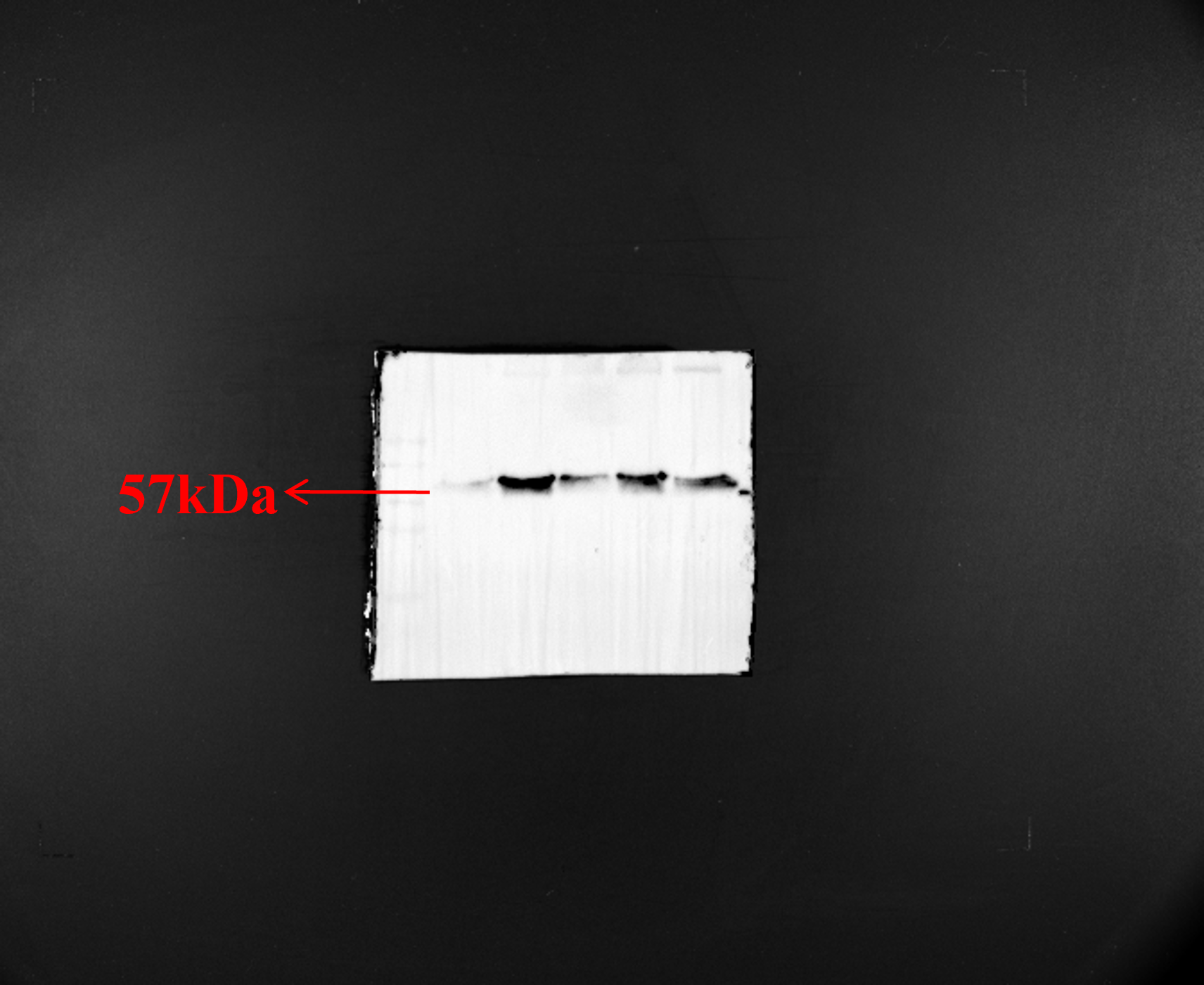

Supplement: Supplementary file 1 [file molecules-30-01173-s001.zip › Figure S8 Fat PPARγ.tif]
